# Using multiple sources during reintroduction of a locally extinct population benefits survival and reproduction of an endangered freshwater fish

**DOI:** 10.1111/eva.13173

**Published:** 2020-12-15

**Authors:** Maiko L. Lutz, Zeb Tonkin, Jian D.L. Yen, Glen Johnson, Brett A. Ingram, Joanne Sharley, Jarod Lyon, David G. Chapple, Paul Sunnucks, Alexandra Pavlova

**Affiliations:** ^1^ School of Biological Sciences Monash University Clayton Vic. Australia; ^2^ Arthur Rylah Institute for Environmental Research (ARI) Heidelberg Vic. Australia; ^3^ School of BioSciences The University of Melbourne Parkville Vic. Australia; ^4^ Department of Environment, Land, Water and Planning Wodonga Vic. Australia; ^5^ Victorian Fisheries Authority Eildon Vic. Australia

**Keywords:** genetic diversity, genetic management, genetic monitoring, multiple source populations, offspring survival, population reintroduction, recruitment, stocking, translocation

## Abstract

Through using different sources, population reintroductions can create genetically diverse populations at low risk of harmful inbreeding and well equipped for adaptation to future environments. Genetic variation from one source can mask locally nonoptimal alleles from another, thereby enhancing adaptive potential and population persistence. We assessed the outcomes in survival, growth and reproduction of using two differentiated sources (genetically diverse Yarra and moderately diverse Dartmouth) for translocations and stocking to reintroduce the endangered Australian freshwater Macquarie perch *Macquaria australasica* into the Ovens River. For stocking, same‐ and different‐population parents (“cross‐types”) were used during hatchery production. Genetic samples and data on individual fish were collected over three years of monitoring the Ovens. We genetically assigned Ovens fish to their broodstock parents and tested whether cross‐type and genetic dissimilarity between parents are associated with offspring survival, and whether cross‐type and parental dissimilarity or individual genetic diversity are associated with somatic growth rates of stocked fish. We genetically identified translocated fish and assessed local recruit ancestry. Of 296 Ovens fish, 31.1% were inferred to be stocked, 1.3% translocated and 67.6% locally born. Cross‐type strongly predicted survival of stocked offspring: those with two Yarra parents had the highest survival, followed by offspring with two‐population, then Dartmouth, ancestry. Of the Ovens recruits, 59.5% had Yarra, 33.5% two‐population and 7.0% Dartmouth ancestry, despite 67% of stocked and 98% of translocated fish originating from Dartmouth. Offspring with two Yarra parents grew faster than offspring of Dartmouth or two‐population ancestry. Although Dartmouth fish appear to be less fit in the Ovens compared to Yarra fish, possibly due to deleterious variation or genetic or plastic maladaptation, they contribute to the reintroduced population through local interbreeding with Yarra fish and relatively high survival of stocked offspring of two‐population ancestry. Thus, combining compatible stocks is likely to benefit restoration of other wildlife populations.

## 1 INTRODUCTION

Human‐driven environmental change has resulted in unprecedented biodiversity loss (IPBES, 2019). Counteracting this through restoration of declining or previously extinct populations requires a multi‐faceted approach, including mitigating the original drivers of decline, considering population sizes, genetic diversity and fit between the current and future environment and genetic composition, morphology, physiology and behaviour of introduced individuals (IUCN/SSC 2013). Small populations experience increased extinction risk due environmental and demographic stochasticity, and loss of genetic diversity, reduced ability to adapt to new conditions, and reduced fitness due to inbreeding (inbreeding depression) (Frankham et al., 2017). Rehabilitated habitat might be suboptimal for individuals translocated from any single source, either because they are adapted to different environments through genetic changes or phenotypic plasticity (e.g. epigenetic modifications experienced during development; Duncan et al., 2014), or because the source population accumulated deleterious variation through genetic drift or relaxed selection (Ferchaud et al., 2018; Huff et al., 2010). Populations established from a single source will usually capture only a subset of the source's genetic diversity (Furlan et al., 2020).

Using multiple populations as sources for reintroduction can increase genetic diversity and the ability to adapt to changing environments and decrease the risk of inbreeding depression in the translocated population, as well as increase the likelihood that some translocated individuals or their descendants will thrive in unpredictable or changing environments (Barrett & Schluter, 2008; Binks et al., 2007). Outcrossing of inbred populations that do not exhibit known predictors of outbreeding depression has overwhelmingly positive fitness effects that extend beyond the F_3_ generation (Frankham, 2015, 2016). Even with noninbred sources, fitness and growth of translocated populations can be elevated by genetic admixture through heterosis, masking deleterious/maladaptive alleles, and rapid adaptation (Binks et al., 2007). Using multiple sources can be appropriate if outbreeding depression (fitness loss due to interbreeding between genetically incompatible or differently adapted populations) and behavioural differences between populations are unlikely, and in the case of uncertainties, adaptive management could alleviate concerns (IUCN/SSC 2013). Gene flow between recently isolated populations that have been evolving in similar environments should have low risk of harming fitness and undermining local adaptation (Frankham et al., 2017). Even with unexpected mating asymmetries or dominance of one stock, mixing distinct populations can still be overall beneficial (Huff et al., 2010; Thavornkanlapachai et al., 2019), although unexpected lack of interbreeding (e.g. due to behavioural differences) will result in lower effective population size than anticipated.

Illustrative cases from a range of wildlife and management scenarios could accelerate the uptake of evolutionary thinking by conservation managers (Cook & Sgrò, 2017). The fitness benefits of using multiple sources for founding populations have been demonstrated in few wildlife species, and monitoring outcomes remains rare (Attard et al., 2016; Thavornkanlapachai et al., 2019; White et al., 2018). Here, we used genetic monitoring to test for the effect of using multiple source populations on survival and recruitment of an endangered freshwater fish, Macquarie perch (*Macquaria australasica* Cuvier 1830) during a reintroduction programme in a south‐eastern Australian river where it had become extinct through human impacts.

The Macquarie perch was once common and widespread through the Murray‐Darling Basin (MDB) of Eastern Australia, but experienced severe population declines and isolation through human impacts (Faulks et al., 2011; Ingram et al., 2000). The attendant population differentiation and very low genetic diversity in most remaining populations makes them vulnerable to inbreeding depression and decreased adaptive potential (Pavlova et al., 2017). The risk of outbreeding depression on mixing populations was assessed to be low, so augmented gene flow was recommended as an urgent management action, adopted by the species‐wide recovery plan (Pavlova et al., 2017; Commonwealth of Australia, 2018).

Macquarie perch became extinct in the Ovens River in the southern MDB in the 1980s, due to excessive recreational fishing, increased sedimentation, habitat degradation and introduction of predatory and competitor fish (Cadwallader, 1981; Trueman, 2011). Over the past two decades, strict fishing regulations have been applied, and major habitat rehabilitation of the Ovens River included livestock removal, restoring riparian revegetation and in‐stream woody habitat, pest control and reintroduction of another extirpated threatened fish species (Department of Environment, Land, Water and Planning, 2017; Lyon et al., 2012).

**FIGURE 1:**
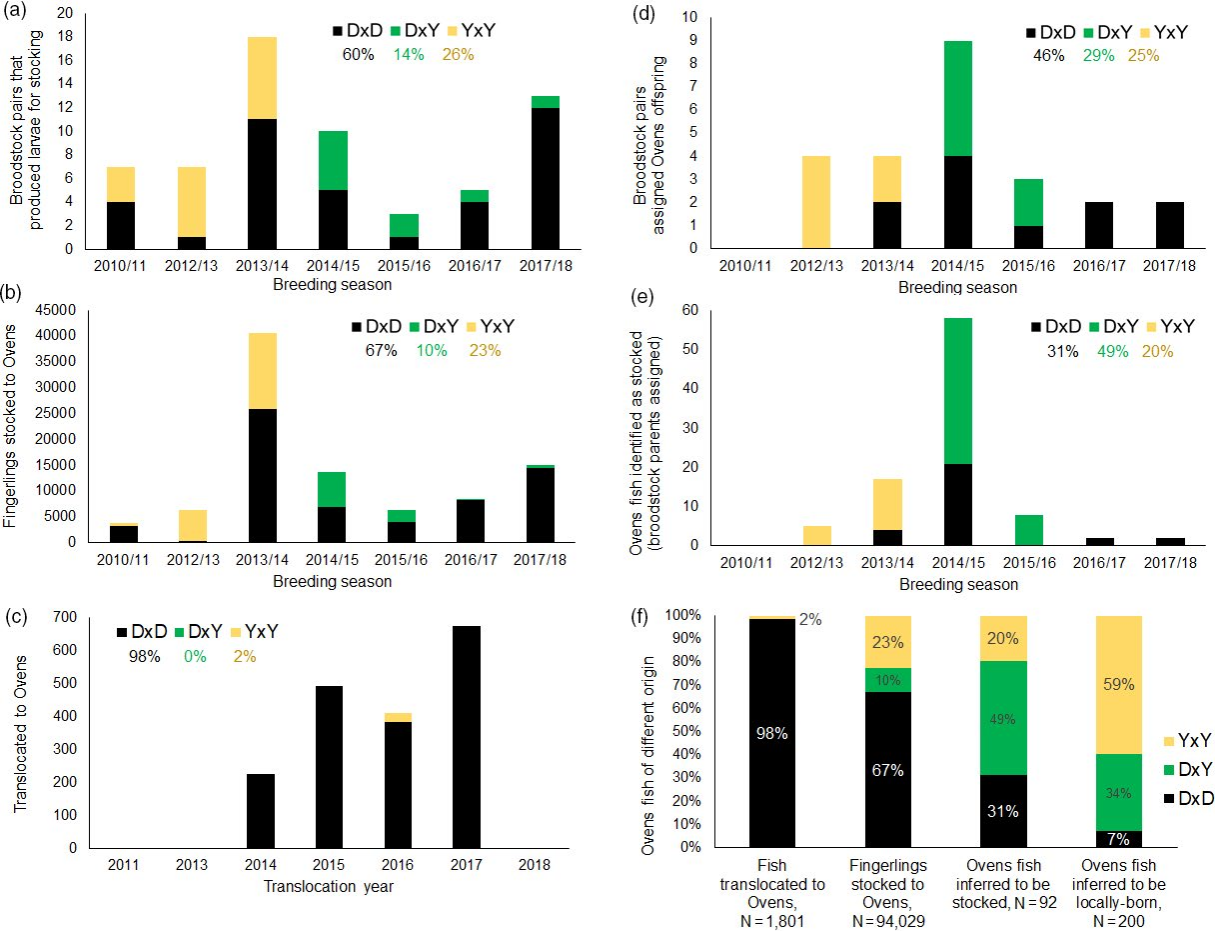
History of stocking and translocation across seven breeding seasons and their outcomes inferred from genetic monitoring for Macquarie perch in the Ovens River: (a) number of broodstock pairs that produced larvae used for stocking, (b) number of fingerlings stocked to the Ovens (estimated based on number of larvae produced by each broodstock pair), (c) number of fish translocated to the Ovens, (d) number of broodstock pairs with offspring detected in the Ovens, (e) number of Ovens fish identified as stocked (i.e. those with broodstock parents assigned to them by genetic parentage analysis) and (f) summary of genetic ancestry for fish that was translocated, stocked, inferred to be stocked, and inferred to be locally born. Colours represent three cross‐types: black—Dartmouth × Dartmouth (DxD), yellow—Yarra × Yarra (YxY), green—Dartmouth × Yarra (DxY). Due to lack of genetic samples for most of 2010/11 broodstock pairs, genetic outcomes for that season should not be interpreted. On (f), 92 Ovens fish inferred to be stocked include 29 DxD + 45 DxY + 18 YxY, and 200 Ovens fish inferred to be locally‐born include 14 DxD + 67DxY + 119 YxY (with a low probability that one DxD and three YxY are stocked offspring of unsampled parents and six DxD are unsampled translocated fish); four translocated individuals identified in the Ovens are not plotted

Macquarie perch reintroduction into the Ovens River began in 2011 with annual stocking of fingerlings produced at the Snobs Creek Hatchery by pairing broodstock males and females from Lake Dartmouth or Yarra River populations. Lake Dartmouth, formed by damming the Mitta Mitta River in 1979, contains the largest remaining MDB population of the species, with fish residing in the lake and extending into the upper Mitta Mitta River and adjacent streams (Tonkin et al., 2014). Although a naturally riverine species, Macquarie perch can occupy reservoirs, given access to suitable riverine habitat for spawning (Cadwallader, 1981). The Yarra River population was established between 1907 and 1943 from multiple southern MDB sources (Cadwallader, 1981; Trueman, 2011) and is the most genetically diverse MDB population, containing unique genetic variation now extinct at the sources (Pavlova et al., 2017). Although Dartmouth and Yarra populations have high genetic diversity and effective population sizes compared to those of other Macquarie perch populations, both can be expected to have compromised ability to adapt to environmental changes, which could be improved through increased genetic variation (Pavlova et al., 2017). Single‐source crosses were used to produce fingerlings for stocking until breeding seasons 2014–2017, when interpopulation (Dartmouth × Yarra) crosses replaced Yarra × Yarra crosses in the conservation breeding programme (Table 1, Figure 1a,b). From 2014 to 2017, stocking was combined with annual translocations of juvenile fish from Lake Dartmouth, which at the time had abundant young fish following the breaking of the “Millennium drought” (Tonkin et al., 2014). Small numbers of adults from Lake Dartmouth, and retired broodstock from both populations, were also released into the Ovens (Table 1, Figure 1c, Figure A2 in Appendix S1).

**TABLE 1:** Summary of management actions for each breeding season/year and inferences of origin (stocked, translocated and locally born) and genetic ancestry (DxD—Dartmouth; DxY—two‐population, YxY—Yarra ancestry) for the Ovens fish captured during monitoring (data in Data S1)

Breeding season		2010/11	2012/13	2013/14	2014/15	2015/16	2016/17	2017/18	Total	% of total
Year of management action		2011	2013	2014	2015	2016	2017	2018		
Translocations										
	All			226	490	413	672		1801	100
	DxD			226	490	383	672		1771	98.33
	DxY			0	0	0	0		0	0.00
	YxY			0	0	27	0		27	1.50
	Unknown			0	0	3	0		3	0.17
Artificial breeding: broodstock pairs that produced larvae										
	All	7	7	18	10	3	5	13	63	100
	DxD	4	1	11	5	1	4	12	38	60.32
	DxY	0	0	0	5	2	1	1	9	14.29
	YxY	2	6	7	0	0	0	0	15	23.81
	Unknown	1	0	0	0	0	0	0	1	1.59
Stocking: fingerlings stocked to Ovens										
	All	3909	6320	40,500	13,600	6400	8300	15,000	94,029	100
	DxD	3216	393	25,895	6967	4045	8175	14,427	63,117	67.13
	DxY	0	0	0	6633	2355	125	573	9687	10.30
	YxY	684	5927	14,605	0	0	0	0	21,216	22.56
	Unknown	9	0	0	0	0	0	0	9	0.01
Monitoring: number of samples obtained										
	All^a^					68	67	167	302	100
Inferred to be stocked	DxD^a^					16	14	16	46	15.23
	DxY^a^					10	3	17	30	9.93
	YxY^a^					7	5	7	19	6.29
Inferred to be translocated	DxD					2	0	2	4	1.32
Inferred to be locally‐born	DxD					6	2	6	14	4.64
	DxY					12	17	38	67	22.19
	YxY^a^					15	26	81	122	40.40
Genetic parentage assignment: number of broodstock pairs assigned offspring										
	All	0	4	4	9	3	2	2	24	100
	DxD	0	0	2	4	1	2	2	11	45.83
	DxY	0	0	0	5	2	0	0	7	29.17
	YxY	0	4	2	0	0	0	0	6	25.00
Genetic parentage assignment: number of Ovens fish inferred to be stocked										
	All	0	5	17	58	8	2	2	92	100
	DxD	0	0	4	21	0	2	2	29	31.52
	DxY	0	0	0	37	8	0	0	45	48.91
	YxY	0	5	13	0	0	0	0	18	19.57
Genetic ancestry assignments for locally born Ovens fish										
	All								200	100
	DxD^b^								14	7.00
	DxY								67	33.50
	YxY^b^								119	59.50

^a^ Three individuals inferred to be stocked (1 DxD, 1 DxY and 1 YxY) and three YxY individuals inferred to be locally‐born were captured twice.

^b^ One DxD and three YxY fish could be stocked offspring of unsampled parents, according to their length/inferred age; six DxD fish have a small chance of being nongenotyped translocated individuals.

Several factors might potentially drive different levels of survival and recruitment in the Ovens River for fish of different ancestry. First, Yarra and Dartmouth Macquarie perch could have phenotypic differences inhibiting free interbreeding (genomic incompatibilities are unlikely, Pavlova et al., 2017). Second, genetically diverse fish descended from multiple stocks may have higher fitness compared to fish of single‐population ancestry due to heterosis, balancing selection or masking of deleterious alleles accumulated in isolated populations (Charlesworth, 2015; Ferchaud et al., 2018). Accordingly, higher fitness could be expected for the offspring of Dartmouth × Yarra pairs, and for offspring of two Yarra parents, themselves descended from multiple diverse stocks. Third, fish from one source might be better suited to conditions in the Ovens (Huff et al., 2010). If this were true, it is more plausible that Macquarie perch from the Yarra River population were better suited to the Ovens than those from Dartmouth, because over the last 40 years (five generations, assuming a generation time of 7 years; Pavlova et al., 2017) Dartmouth fish have spent most of every year in lacustrine environments. Here, they could have experienced plastic changes, relaxed selection for riverine conditions and/or positive selection for lake conditions, potentially making them less suited to the Ovens River in only a few generations (Bell et al., 2004; Duncan et al., 2014; Lahti et al., 2009). Adaptations to stream environments have not been assessed for Macquarie perch, but are known in fishes (Bunt et al., 2004).

Here, we use genotypic data from reduced‐representation genome‐wide markers in genetic analyses including identity, parentage, kinship and genetic cluster assignment and hybrid analysis. These analyses were applied to identify, among individuals captured during annual monitoring in the Ovens River (2016–2018), hatchery‐produced offspring, translocated fish and offspring of translocated fish, and to assign genetic ancestry to local recruits. We use these data to answer the following questions: (a) Do offspring of different genetic ancestry (i.e. of cross‐types involving Dartmouth‐only, Yarra‐only or two‐population parents) and more genetically dissimilar parents have higher survival following stocking? (b) Are genetic ancestry, and individual genetic diversity or parental dissimilarity, associated with individual growth rates? (c) Do fish of different genetic ancestry have different levels of recruitment in the Ovens? (d) Do Yarra and Dartmouth fish freely interbreed in the wild?

By using a nonlethal genetic method of monitoring stocking and translocation success in a wild population, our study provides rare information on growth and survival of stocked fish, local recruitment, and fitness outcomes when multiple source populations are used to reintroduce an extinct population.

## 2 MATERIALS AND METHODS

### 2.1 Study system: artificial breeding, stocking and translocations to the Ovens River

Artificial breeding of Macquarie perch was performed at the Snobs Creek Hatchery using established methods (Ho & Ingram, 2013). Broodstock were collected as “running ripe” fish from the Yarra River, and from Lake Dartmouth (captured during their annual temporary spawning migration into the Mitta Mitta River), in November–December of 2010–2017 (austral spring—summer). Eggs from each female were mixed with milt of one male and incubated separately; the number of hatched larvae was estimated before pooling (Table A1 in Appendix S1). Pooled larvae were grown to fingerlings (0.5–1 g) in fertilized earthen ponds. Fingerlings harvested from ponds were released the following January–February across 10 sites along a ~50 km stretch of the Ovens (>94,000 in total; Figure 1b, Table A2 in Appendix S1; Figure A1 in Appendix S1).

A total of 1801 fish were also translocated to the Ovens from 2014 to 2017 (Table A3 in Appendix S1), including wild‐caught juveniles (<3‐year old) and adults from Dartmouth (89.1% and 19.9%, respectively; *N* = 1739; Figure A2 in Appendix S1) and retired broodstock from Dartmouth (*N* = 32), Yarra (*N* = 27) and of unknown origin (*N* = 3). Fin‐clip tissue samples for DNA analyses were collected from 98/108 broodstock (Table A1 in Appendix S1) and 1770/1801 translocated fish, and stored in absolute ethanol at −20°C.

### 2.2 Fish monitoring

Monitoring surveys were conducted at multiple sites in the Ovens in February–May in 2016, 2017 and 2018, resulting in 68, 67 and 167 Macquarie perch captured, respectively (Table A4, Figure A1 in Appendix S1). These sites encompassed all stocking and translocation release sites, plus upstream and downstream of them. Fish were caught using boat‐mounted electrofishing equipment (Smith‐Root® Model 5 GPP), measured for total length (nearest mm) and weight (nearest g), fin‐clipped and released at their capture site.

### 2.3 Sampling, DNA extraction, genotyping and data filtering

A total of 2237 Macquarie perch samples genotyped encompassed all 302 fish captured during Ovens monitoring, 71 Dartmouth and 27 Yarra Snobs Creek hatchery broodstock parents that yielded fingerlings stocked to the Ovens (of which 14 Dartmouth and 10 Yarra were later released to the Ovens; Table A1 in Appendix S1), 1727 Dartmouth and 19 Yarra additional fish translocated to the Ovens, 30 hatchery‐produced fish with known parents, and 17 Dartmouth and 44 Yarra reference fish not released to the Ovens (Data S1). Of a total 1801 Macquarie perch translocated to the Ovens, 98.3% were genotyped (1741 Dartmouth, 29 Yarra); and 1.7% (29 Dartmouth, two unknown) were not.

DNA was extracted from fin‐clips using a DNeasy Blood and Tissue Kit (Qiagen). Genotyping and single nucleotide polymorphism (SNP) calling were performed at Diversity Arrays Technology Pty. Ltd., using reduced‐representation sequencing DartSeq™ protocols (Kilian et al., 2012), following Nguyen et al. (2018). SNP data were filtered using the dartR package (Gruber et al., 2018) in R (R Core Team, 2019). Unless otherwise stated, a single biallelic SNP per locus with reproducibility = 1 (estimated based on re‐processing of ~25% samples) was retained, loci missing scores in >10% of individuals and 41 individuals missing >25% of genotypes were removed (Data S1; these were fish translocated from Dartmouth, including 27 that were mature in 2017). To ensure that loci with a signal of hemizygosity in one sex are excluded from analyses that assume codominant markers, the loci were tested for sex linkage in a panel of known‐sex broodstock fish using the gl.sexlinkage dartR function: none were homozygous in one sex and heterozygous in the other. Different numbers of polymorphic loci were retained after removing monomorphic loci for various subsets of individuals (see below).

### 2.4 Parentage, sibship and identity analyses

Data for 1679 individuals scored for 1204 loci were used for parentage analysis in CERVUS3 (Kalinowski et al., 2007) with error rate of 0.0001 (justification and details in Appendix B in Appendix S1, including calibration on known‐parentage offspring). All Ovens fish were used as candidate offspring. Successful broodstock and translocated fish of breeding age (>2 years) in December 2017, representing potential parents of fish captured during the last round of monitoring in 2018, were used as candidate parents. We assumed that 70% of parents were sampled and 90% of loci genotyped. Simulations to calibrate likelihood thresholds for maternity and paternity assignments were run using 250 potential parents and 100,000 offspring.

COLONY2 (Jones & Wang, 2010) was used to conduct sibship and parentage analyses using the same dataset, assuming a polygamous, noninbreeding, dioecious diploid mating system, with a locus error rate of 0.0001 and 70% probability of parents being included. Medium run length and full likelihood were used to estimate precision. We used the option to calculate allele frequencies but not to update allele frequencies after calculations, because family sizes and structure were unknown. Sibship scaling was applied.

Only parent‐offspring pairs assigned with probability >95%, and full‐sibship groups assigned with probability >98%, were accepted. Ovens individuals assigned as offspring of two broodstock parents from the breeding programme were inferred to be stocked, and their birth year and age at sampling calculated based on the year their parents were mated, assuming hatching on 1st of December.

To detect translocated fish, individuals sampled more than once, and potential mislabelling errors, we ran identity analysis in CERVUS3 for 2173 individuals: all Ovens fish, all genotyped fish that were translocated to the Ovens, and all broodstock fish. Loci with repeatability <1 and those missing scores in <10% of individuals were removed for this analysis; 1120 loci were retained. Fuzzy matching with up to 50 mismatched loci was applied; two samples were considered identical if they matched for >99% of their allele scores.

### 2.5 Individual genetic diversity and genetic dissimilarity between broodstock parents

For 92 fish inferred as stocked (based on parentage, above), individual genetic diversity, expressed as proportion of heterozygous loci (PHt), was calculated using GENHET in R (Coulon, 2010) from 735 loci. Genetic dissimilarity between members of a parent pair, expressed as the proportion of allelic differences between male and female genotypes, was calculated for 54 genotyped broodstock pairs that produced larvae, thus presumed to have yielded fingerlings stocked to the Ovens. The diss.dist function in R (Kamvar et al., 2014) was used on 938 loci scored for 91 broodstock fish.

### 2.6 Modelling relative offspring survival after stocking

We assessed whether cross‐type (i.e. one of three types of broodstock crosses: Dartmouth × Dartmouth, denoted DxD below, Yarra × Yarra, YxY, and Dartmouth × Yarra, DxY) and genetic dissimilarity between broodstock parents can predict poststocking offspring survival. The latter was approximated for broodstock parent‐pairs bred over six consecutive breeding seasons (2012/2013–2017/2018; details in Appendix C in Appendix S1) using capture rates of Ovens individuals assigned as broodstock offspring, for each year of their life. Our data for Dartmouth fish captured using the same approach suggested no age‐based capture biases, except that very young offspring (<1‐year old) were much less likely to be captured than older fish (≥1‐year old; Figure A2 in Appendix S1).

First, for each of the 53 unique broodstock pairs, the minimum number of surviving offspring was calculated for each of six age classes (from zero to the maximum of 5 years old, for fish stocked in 2013), based on the number of offspring assigned to each pair by parentage analysis for each year of sampling (from 2016 to 2018; Data S2). Offspring sampled in a particular age class were assumed present in all previous age classes. Next, the relative probability of offspring survival for each age class was estimated as the minimum number of surviving offspring for each age class divided by the number of stocked fingerlings produced by each pair (Table A1 in Appendix S1; estimated as the number of larvae produced by that pair divided by the total number of larvae produced that year by all broodstock fish, multiplied by the total number of fingerlings stocked that year into the Ovens).

We used generalized linear mixed models to relate the proportion of surviving offspring to parental cross‐type (DxD, DxY, YxY) and genetic dissimilarity between broodstock parents (Appendix C in Appendix S1). We modelled proportional offspring survival as a binomial variable with logit link, with weights defined as the number of stocked fingerlings. We included year of stocking as a random effect to account for repeated measures on each cohort, as well as variation in survival due to environmental factors (e.g. streamflow, level of predation, population density or nutrient availability soon after stocking). We fitted separate models with each of cross‐type and parental genetic dissimilarity, as well as an additive model including both. Because probability of fish survival increases with age and probability of detection differs between age classes, we also fitted these same models with an interaction between age and both fixed effects. We fitted all models with the glmer function in the R package lme4 (Bates et al., 2015) and compared models using Akaike's Information Criterion (AIC). We assessed model fit with pseudo‐*R* ^2^ values calculated using the R package MuMIn (Barton, 2015) (Appendix C in Appendix S1).

### 2.7 Modelling individual growth

For the 92 Ovens fish identified as stocked, we tested whether growth varied with cross‐type, and either individual genetic diversity or parental dissimilarity. We fitted a standard Gompertz nonlinear regression model to length and age of Ovens fish identified as stocked and calculated residuals, reflecting the difference in their growth compared to the model average. These residuals were used as the response variable in linear mixed models in which cross‐type (DxD, DxY, YxY), and genetic diversity or genetic dissimilarity between broodstock parents was fitted as fixed effects (Data S2; details in Appendix D in Appendix S1). We fitted models with all three predictors separately, as well as additive models of cross‐type with genetic diversity or genetic dissimilarity, and used AIC values to identify the most parsimonious model structure (Appendix D in Appendix S1). We included year of stocking as a random effect in all models. We also used this Gompertz model to estimate age of Ovens fish unassigned to broodstock parents (i.e. inferred to be nonstocked), from their length.

### 2.8 Population structure and recruitment

To investigate the possibility of local interbreeding between Dartmouth and Yarra fish, we assessed genetic ancestry of Ovens fish inferred to be locally‐born (i.e. not assigned as stocked or translocated). Starting with a dataset for 564 individuals scored for 1003 loci (single SNP per locus with repeatability = 1) with <5% missing data, we calculated per locus *F*_ST_ values between Dartmouth and Yarra samples of randomly‐chosen individuals (51 per sample, not broodstock parents) using the R package diversity (Keenan et al., 2013). Then, we used the 407 loci most differentiated between Dartmouth and Yarra (*F*_ST_ ≥ 0.02) in STRUCTURE analysis (Pritchard et al., 2000) to assign membership to two genetic clusters (*K* = 2, for Dartmouth and Yarra) for 157 unrelated Ovens fish inferred to be nonstocked (excluding all but one full‐sibling detected by COLONY2), 23 similarly unrelated fish inferred to be stocked (with known ancestry), and 51 each from Dartmouth and Yarra (as above; 282 total). The sibling with the lowest level of missing data within each full‐sibling group was used to represent its group. Twenty STRUCTURE replicates were run using 10^6^ burnin and 10^6^ MCMC samples; these replicates were summarized using CLUMPAK (Kopelman et al., 2015). For each Ovens fish of unknown ancestry included in the STRUCTURE analysis, we assigned Dartmouth, two‐population or Yarra ancestry based on Q‐value thresholds estimated for individuals of known Dartmouth or Yarra ancestry. For the full‐siblings excluded from STRUCTURE, ancestry was assigned based on that of their included sibling. To test for local recruitment of DxY fish, we assigned locally born Ovens individuals to six ancestry classes (two sources, F_1_, F_2_, and two backcross types; details in Appendix B in Appendix S1) using NEWHYBRIDS (Anderson & Thompson, 2002).

## 3 RESULTS

### 3.1 Parentage, identity and sibship analyses

Identity analysis revealed that among the fish sampled during monitoring of the Ovens River, six individuals were sampled in two monitoring seasons, and four had been translocated from Dartmouth (Appendix B, Table B2 in Appendix S1; seven sample mislabelling errors were also detected, which is 0.3% of 2173 fish analysed for identity). A total of 92 of 296 unique Ovens individuals (31%) were assigned both parents among known broodstock parent pairs and were thus inferred to be stocked (Figure 1e, Appendix B, Table B1 in Appendix S1). Of the 54 genetically sampled broodstock pairs that contributed to Ovens stocking (Table A1 in Appendix S1), 24 (44%) were assigned as parents of Ovens individuals sampled during monitoring (Table B1 in Appendix S1, Figure 1d), with up to 16 offspring per pair detected (median of 2.5 for parent‐pairs assigned offspring). In addition, four Ovens fish were assigned both parents among translocated Dartmouth fish, supporting local recruitment (Appendix B in Appendix S1). Forty full‐sibship groups were detected by COLONY2 (Table B3 in Appendix S1; Data S1).

### 3.2 Contribution of different years of the breeding programme, types of broodstock crosses and parental dissimilarity to relative survival of stocked offspring

More than 90% of 92 Ovens fish identified as stocked were born in November/December 2013, 2014 and 2015 (17, 53 and 13 individuals, respectively; Figure 1e), while only 64% of all fingerlings were released over those three years (40,500, 13,600 and 6400, respectively; Figure 1b). This suggests particularly good survival in some years.

**FIGURE 2:**
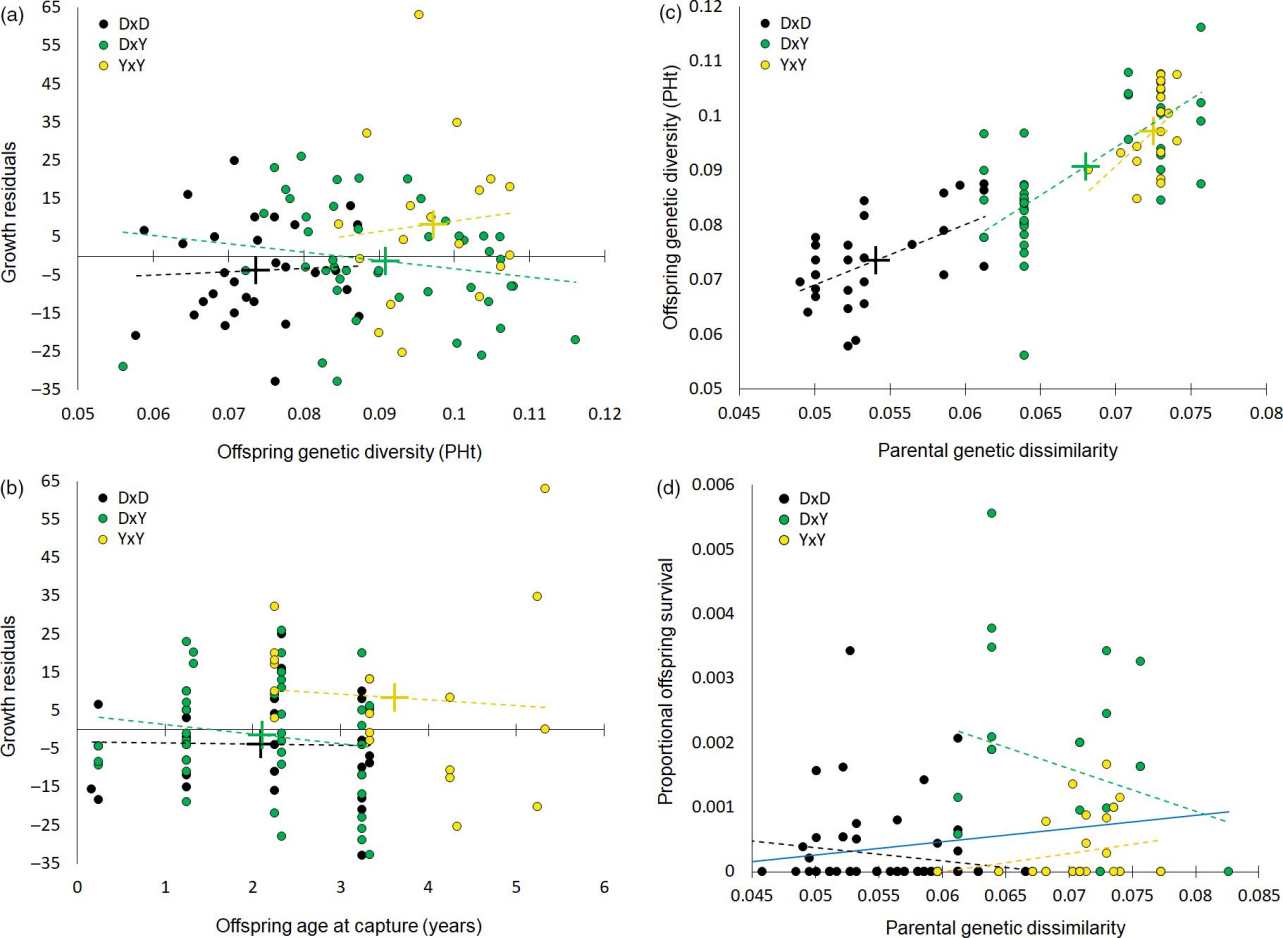
Variation in growth, age at sampling, genetic diversity, parental genetic dissimilarities and inferred relative offspring survival among three broodstock cross‐types: black—Dartmouth × Dartmouth (DxD), yellow—Yarra × Yarra (YxY), green—Dartmouth × Yarra (DxY): (a) growth residuals plotted against genetic diversity (PHt) and (b) against age at sampling for each Ovens fish inferred to be stocked; (c) offspring genetic diversity plotted against parental genetic dissimilarity for each Ovens fish inferred to be stocked; (d) raw values of proportional offspring survival (the number of observed offspring of each age class divided by number of stocked fingerlings) plotted against parental genetic dissimilarity for each broodstock pair that contributed offspring to stocking; for each cross‐type, linear trends are shown as black (DxD), yellow (YxY) or green (DxY) dashed lines, and means as crosses; the blue line shows a linear trend for offspring survival for all broodstock pairs combined. Note that (d) does not account for year of stocking, inclusion of offspring of the same pair sampled multiple times at different ages and decreased probability of offspring survival with age (this is done in the survival model; Table 2)

Among the Ovens fish identified as stocked, 31% were offspring of DxD crosses, 20% YxY and 49% DxY (Figure 1e, Table 1 and Table B1 in Appendix S1), despite respective contribution of stocked fingerlings being 67% DxD, 23% YxY and 10% DxY (Figure 1b). For the Ovens fish identified as stocked, mean parental genetic dissimilarity and mean offspring genetic diversity were low in DxD, intermediate in DxY and high in YxY offspring, with variance for both genetic variables being low in DxD, intermediate in YxY and high in DxY offspring (Figure 2c; mean ± *SD* genetic dissimilarity = 0.054 ± 0.004 for DxD, 0.068 ± 0.005 DxY and 0.072 ± 0.001 YxY; PHt = 0.074 ± 0.008 DxD, 0.091 ± 0.012 DxY and 0.097 ± 0.007 YxY).

The most parsimonious model of survival included all predictor variables (parental cross‐type, genetic dissimilarity between parents, offspring age class; Appendix C, Table C2 in Appendix S1). This model explained 40% of variation in relative offspring survival to all ages (Table 2). Parental cross‐type and genetic dissimilarity were significant predictors of offspring survival, with survival highest in offspring of YxY pairs, followed by DxY, then DxD (Table 2). That is, cross‐types characterized by the greater parental dissimilarity had better‐surviving offspring. In addition to this main effect, there was a weak residual pattern that within cross‐types, offspring from more genetically dissimilar pairs had slightly lower survival than those from less genetically dissimilar pairs (Table 2). However, this effect was inconsistent among cross‐types (Figure 2d, Appendix C in Appendix S1). Although survival qualitatively decreased with age, there was no significant effect of offspring age class. An interaction term between age and cross‐type and parental genetic dissimilarity was not significant, suggesting that all age classes had similar associations with cross‐type and dissimilarity, despite age class 0 being over‐represented through being inferred from captures when they were older fish. The ranking of models based on AIC and significance of cross‐type and parental genetic dissimilarity as predictors of offspring survival did not change when YxY pairs were removed from the analysis (Tables C2 and C3 in Appendix S1), indicating that higher survival of YxY offspring was not driving the significant result.

**TABLE 2:** Results of the most parsimonious offspring survival model

Variables	Cross‐type	Coefficient	Standard error	Pr(>|*z*|)	R2m
Intercept		−8.09	0.48	**<0.001**	0.40
Age class		−0.22	0.12	0.063	
Genetic dissimilarity		−0.43	0.18	**0.020**	
Cross‐type	DY	1.62	0.35	**<0.001**	
	YY	2.28	0.48	**<0.001**	
Age class × genetic dissimilarity		0.04	0.10	0.67	
Age class × cross‐type	DY	–0.15	0.19	0.45	
	YY	0.01	0.22	0.97	

Note

Probability of offspring survival after stocking to ages of <6 months, 1‐year old, 2‐year old and 3‐year old, as a function of offspring age class, parental cross‐type and genetic dissimilarity between members of a broodstock parent pair. This model included all age classes, with year of stocking fitted as a random factor to account for repeated measures of each cohort (see Appendix C in Appendix S1 for results of model comparisons and additional models). R2m is a likelihood ratio‐based pseudo *R* ^2^ measure based on theoretically derived binomial variances. Significant *p*‐values are in bold.

### 3.3 Growth of Ovens fish identified as stocked

Parental cross‐type (genetic ancestry) was the only predictor included in the most parsimonious model of offspring growth (lowest AIC; Appendix D, Table D1 in Appendix S1). Models including cross‐type and offspring genetic diversity (PHt) or parental genetic dissimilarity both had ΔAIC ≤ 2 (Table D1 in Appendix S1). All three models explained ~7% of the variation in residual growth, with cross‐type being the only significant predictor of relative growth (Table D2 in Appendix S1). The offspring of YxY crosses stocked into the Ovens were bigger than DxD and DxY ones of the same age (Figure 2b). However, YxY and DxY crosses were not performed simultaneously (Figure 1a), thus this comparison should be interpreted with caution. No other variables were significantly associated with offspring growth (Figure 2a, Table D2 in Appendix S1).

### 3.4 Genetic ancestry of Ovens fish inferred to be locally born

**FIGURE 3:**
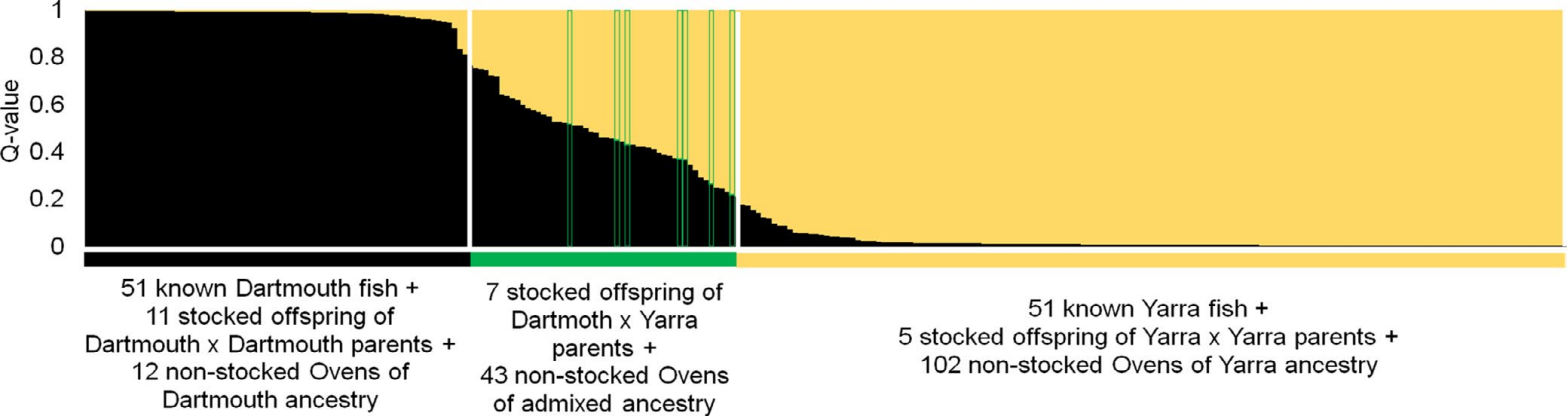
Results of STRUCTURE analysis, built from loci with *F*_ST_ ≥ 0.02 between Dartmouth and Yarra, for unrelated Ovens fish and fish from Dartmouth and Yarra (*N* = 51 each). Membership of each individual in the black (Dartmouth, *Q*_D_) and yellow (Yarra, *Q*_Y_) genetic clusters is indicated by thin vertical lines, individuals are arranged by *Q*_D_. White vertical lines indicate empirical limits of membership of the Dartmouth cluster for Dartmouth fish (*Q*_D_ > 0.76) and for Yarra fish (*Q*_D_ < 0.22), based on individuals of known ancestry included in the analysis. Known Dartmouth × Yarra offspring (one per full‐sibship group, marked with thin green border) had 0.52 > *Q*_D_ > 0.22. Coloured bars below the plot summarize the categories of individuals known to belong to each group, along with the genetic ancestry of locally born Ovens fish inferred from these known individuals. All Ovens fish with 0.76 > *Q*_D_ > 0.22 were assigned two‐population ancestry (Dartmouth × Yarra or backcrosses; green bar below the plot)

**FIGURE 4:**
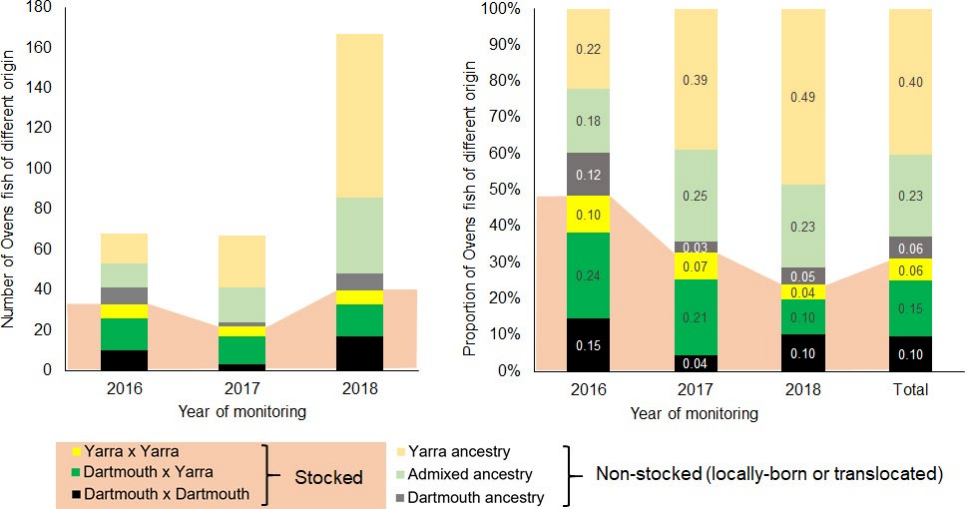
Number (left) and proportion (right) of the Ovens fish inferred to be stocked and nonstocked (i.e. locally born and translocated), sampled across 3 years of monitoring, with their genetic ancestry (inferred from parentage analysis for stocked, and from STRUCTURE and full‐sibship analyses for locally born)

Whereas of 296 unique Ovens individuals 92 (31.1%) were identified as stocked and four (1.4%) as translocated from Dartmouth (above), the remaining 200 (67.6%) were inferred to be locally‐born (including four identified as DxD offspring of two translocated parents), although four of these 200 (three YxY and one DxD) were old enough to have been stocked in one of the three earliest seasons when one or more broodstock parents were not sampled (Table A1 in Appendix S1) and six DxD individuals were the same age as some unsampled translocated fish (below; Table 1). Known Dartmouth, DxY and Yarra fish clustered separately in the STRUCTURE analysis, according to decreasing membership to the Dartmouth genetic cluster (Figure 3): all fish of known Dartmouth origin had membership to the Dartmouth genetic cluster *Q*_D_ > 0.76, and all fish of known Yarra origin had *Q*_D_ < 0.22; stocked DxY fish had 0.22 < *Q*_D _< 0.52. This enabled assignment of 9, 25 and 102 unrelated Ovens fish inferred to be locally‐born into three groups according to their Dartmouth/two‐population/Yarra ancestry, respectively, with 18 additional individuals also showing levels of two‐population ancestry falling between those of observed Dartmouth and DxY individuals. Assigning the remaining locally born siblings to the same origin as their analysed sibling resulted in 14 unique locally born fish being assigned to Dartmouth, 67 to two‐population and 119 to Yarra ancestry (Table 1, Figure 4; data in Data S1). NEWHYBRIDS results were generally consistent with STRUCTURE and further assigned the 67 two‐population hybrids to 65 F_1_s and 2 backcrosses to Yarra (details in Appendix B in Appendix S1).

Of 14 Ovens fish of Dartmouth origin, seven were unambiguously local recruits: four were assigned parents among fish translocated from Dartmouth (with inferred birth dates postdating translocation date of the parent), and three were too small to have been nongenotyped translocated fish. Of the remaining seven fish of Dartmouth ancestry, one was old enough to potentially represent stocked offspring of unsampled broodstock pairs, and six (of which three are full‐siblings) have <2% chance of being nongenotyped translocated individuals. Because all broodstock parents that produced DxY fingerlings were sampled, the 67 fish of two‐population ancestry not assigned broodstock parents necessarily come from local interbreeding between fish of Dartmouth and Yarra origin, or offspring of stocked DxY individuals (including back‐crosses to Dartmouth and Yarra fish). Two fish of two‐population ancestry inferred to be born before the start of translocations in 2014 (Data S1) are likely offspring of two stocked fish (one of Dartmouth and one of Yarra ancestry). Of the 119 putatively locally‐born Ovens fish of Yarra origin, three were old enough to be stocked offspring of unsampled broodstock pairs, whereas the remaining 116 were too young for that, so must be local recruits (Table D4 in Appendix S1).

The proportion of Ovens fish inferred to be locally born of two‐population and Yarra ancestry generally increased across years of monitoring due to an increase in the proportion of locally recruited fish, comprising 0.40, 0.64 and 0.71 of the total monitoring sample in 2016, 2017 and 2018, respectively (Figure 4). In contrast, the contribution of fish inferred to be locally born and translocated of Dartmouth ancestry to the respective monitoring samples was only 0.12, 0.03 and 0.05 (Figure 4), with only 7% (14 individuals) of 200 fish inferred to be locally born in the Ovens being of Dartmouth ancestry, despite 98% of translocated fish being from Dartmouth.

## 4 DISCUSSION

We aimed to test whether using multiple source populations for stocking and translocation during population reintroduction benefits survival and recruitment of fish in the wild. A third of Macquarie perch captured in the Ovens River were inferred to be stocked, using genomic assignment to broodstock parents. For these hatchery‐produced fish, parental cross‐type was the most important predictor of survival (approximated here by capture rates per year of offspring life) and growth. Offspring of Yarra × Yarra crosses, followed by Dartmouth × Yarra crosses, had a higher chance of reaching maturity, having higher survival for the first years of life, with offspring of Dartmouth × Dartmouth crosses having lowest survival. It is possible that, regardless of their genetic diversity, approximated in our survival models by parental dissimilarity, offspring of Yarra ancestry are fitter in the Ovens River than those of Dartmouth ancestry, consistent with offspring of Yarra × Yarra crosses having the highest relative growth. The results of powerful individual‐based genetic analyses, including genetic ancestry assignment of each Ovens fish inferred to be locally‐born, together with analysis of fish age, further supports this: local recruits in the Ovens River population mostly comprise fish of Yarra and two‐population ancestry (Figure 4). No negative fitness outcomes of using two source populations were observed: the abundance of locally born F_1_ Dartmouth × Yarra hybrids in the Ovens River shows that Yarra and Dartmouth Macquarie perch can successfully interbreed. Presence of backcrosses to Yarra, inferred to be born in 2016 (Figure 3, Appendix B in Appendix S1), further supports local recruitment of locally‐born DxY fish. Given that some Macquarie perch of each sex can reach maturity by 3 years of age, fish of Yarra and Dartmouth ancestry stocked in 2011 could have interbred in the Ovens from 2013/14, and backcrossed from 2016/17, whereas first hatchery‐produced DxY offspring were stocked in 2015 and could have backcrossed only from 2017/18. Future genetic monitoring and local recruit analysis will be required to understand fitness of locally born F_2_ and backcrosses. The contribution to the Ovens monitoring sample of fish translocated from Dartmouth was surprisingly low (2.6% including translocated fish and offspring of two translocated parents), although we could not distinguish recruits of stocked fish from those of one stocked and one translocated parent. Whatever the underlying causes of relatively high fitness of fish with two‐population ancestry (and acknowledging that multiple drivers are not mutually‐exclusive), our study demonstrates positive effects under natural conditions of crossing recently diverged stocks of an endangered wildlife species. The Ovens Macquarie perch example adds to the growing list of cases that should encourage managers of other species with fragmented ranges to consider using multiple sources for improving population persistence.

In our survival models, offspring survival was approximated by capture rates per year of life, where captured offspring were assumed to be present in earlier age classes. Because very young fish (<1‐year old) were less likely to be captured during monitoring (Figure A2 in Appendix S1), the first age class in our survival analysis was strongly influenced by captures of older fish inferred to be alive at age‐0. Our assumption should not have strongly biased relative survival towards the presence of more YxY and DxY offspring, because YxY and DxY cross‐types were less abundant in stocked fish than were DxD cross‐type for all but one breeding season (Figure 1b). The exception was 2012/13 breeding season, in which 94% of all stocked offspring were estimated to be YxY (Table 1) and five offspring of four YxY pairs were captured during monitoring (Table B2 in Appendix S1). Nevertheless, our offspring survival model yielded the same result even when run without YxY pairs (Table C3 in Appendix S1). Moreover, the nonsignificant age interaction term suggested that the relationships between capture rates and cross‐type and genetic dissimilarity were similar across all age classes. It is unlikely that very small variation in size within each age class (Figure 2b) biased capture rates in a way that could have impacted our analyses. Only YxY fish were inferred to be significantly larger than DxY or DxD fish of the same age, and yet our analysis yielded higher survival for DxY (as well as YxY) fish, compared to DxD fish. Our data also do not support lower detection probability of DxD fish due to their preference for less sampled habitats within the Ovens River: stocked DxD offspring were sampled at 54.8% of all capture locations of stocked offspring (17/31), and at majority of these (11/17) other cross‐types were also sampled (data not shown).

According to theory and empirical evidence, augmenting genetic diversity through mixing suitable stocks can improve population survival and adaptive potential (Frankham et al., 2017; Weeks et al., 2015). As such, the Yarra population of Macquarie perch, founded early in the 20th century by fish from various tributaries of the Murray River, represents a successful example. Given relatively high genetic diversity of the Yarra and Dartmouth populations, reintroducing from two sources into the Ovens was intended mainly to enhance population adaptive potential, not so much to alleviate inbreeding depression, although higher fitness of offspring of two‐population and Yarra ancestry could be expected under heterosis, masking deleterious alleles or balancing selection (Charlesworth, 2015; Ferchaud et al., 2018). Our findings of disproportionately many local recruits and superior survival of stocked offspring of Yarra × Yarra, followed by Dartmouth × Yarra cross‐types (Figure 1f, Table 2) count against Dartmouth × Yarra heterosis, but support, as potential drivers, balancing selection associated with Yarra variants, or masking deleterious alleles accumulated in Lake Dartmouth. Cross‐type had a strong association with survival of stocked offspring, in the expected direction of greater survival with greater interparental dissimilarity. In comparison, a negative within‐cross‐type effect of dissimilarity was very weak (explanation in Appendix C in Appendix S1) and inconsistent across cross‐types (i.e. positive trend for YxY offspring), and for DxY, the effect was amplified by a single most dissimilar pair with no sampled offspring (Figure 2d), suggesting it could represent noise.

On the other hand, higher fitness of Yarra‐ than Dartmouth‐lineage fish in the Ovens, associated with differences between riverine and lacustrine environments, could also explain superior survival and recruitment of fish of Yarra‐ and two‐population ancestry. The Dartmouth fish for broodstock and translocations were collected from Lake Dartmouth or during annual spawning migrations into its main tributary, the Mitta Mitta River, immediately upstream. Hence, all or most of these fish are likely Lake residents, accessing riverine environments only for spawning (Tonkin et al., 2018). Although fish experience similar spring spawning temperatures and flow variability across populations (Tonkin, Kearns, Fanson, et al., 2017; Tonkin, Kearns, Lyon, et al., 2017), the conditions experienced by fish during summer are likely very different. Unlike the lacustrine population, riverine populations often experience low flow, extreme habitat contractions, prolonged exposure to high water temperatures and likely, low food availability (Tonkin et al., 2014, 2019). These stressful conditions regulate population dynamics (Tonkin et al., 2019) and impose strong selection pressures on riverine Macquarie perch populations, which could have resulted in adaptation to extreme habitat conditions and/or better purging of deleterious mutations, potentially making Yarra fish fitter. Conversely, Lake Dartmouth, being a large and deep reservoir, provides stable habitat, temperature ranges and, presumably, food resources (Hunt et al., 2011). Thus, through loss of adaptation to extreme summer conditions, adaptation to lake environments and/or plasticity, Dartmouth fish may be less suitable for translocation to riverine habitats than are fish from the Yarra River. Relaxed selection pressures in the lake for stressful summer conditions, especially during Macquarie perch population growth in Lake Dartmouth after initial filling and refilling after a prolonged drought (Tonkin et al., 2014), could have promoted accumulation of mutations suboptimal in riverine environments, resulting in relatively poor performance of Dartmouth fish translocated to the Ovens River (Figure 4). Loss of adaptive traits under relaxed selection and gain of locally adaptive traits can manifest in fish after a few generations (Bell et al., 2004; Fraser et al., 2011; Lahti et al., 2009), and there is increasing evidence that environmental effects on life history can be transgenerational (Burton & Metcalfe, 2014). Because lake environments were available for Dartmouth fish for 40 years, translocated fish could have been evolving under these conditions for up to five Macquarie perch generations.

Poor performance of fish moved from lakes to rivers is not necessarily expected: in other species, such as burbot *Lota lota*, progeny from lacustrine broodstock survive, grow, disperse and spawn in a riverine environment (e.g. Hardy et al., 2015). Importantly, even though fish of Dartmouth ancestry had poor recruitment and growth relative to fish of Yarra ancestry, they still made a considerable genetic contribution through breeding with fish of Yarra ancestry, and the most heterozygous individuals in the Ovens sample were of two‐population ancestry. Thus, the presence of any human‐driven local adaptation in source populations should not necessarily prevent using multiple sources for fear of introducing maladaptation, especially if the size of a target population is large enough for selection to overcome genetic drift. Indeed, gene flow from a differently adapted population can cause genetic and demographic rescue of small populations without swamping locally adapted alleles (Fitzpatrick et al., 2020).

The strong contribution of Yarra fish to the restored Ovens population suggests great genetic value of the Yarra population for future survival and restoration of Macquarie perch. This may be through Yarra fish not possessing unfavourable alleles that are present in Dartmouth, or because they have adaptive variation that is absent in Dartmouth. Because many of its sources are now extinct, the Yarra population contains unique genetic diversity that might assist future adaptation of the species to changing environments (Pavlova et al., 2017). Very few broodstock fish could now be sourced from the Yarra due to a decline of large adults, likely contributed to by overfishing (Tonkin, Kearns, Fanson, et al., 2017). Our findings here support the recent close to the recreational fishery in the Yarra, due to its importance for conservation management of the species (Pavlova et al., 2017; Tonkin, Kearns, Fanson, et al., 2017; Tonkin et al., 2019). Attempts to recover and augment Macquarie perch populations through stocking of hatchery‐bred fish and translocation of wild ones have been undertaken intermittently since the 1980s with mixed success (Lintermans, 2013; Commonwealth of Australia, 2018). The crucial role of Yarra source in the success of the Ovens population rehabilitation suggests that restoration efforts involving movement of fish from impoundments into riverine populations might be strengthened by additionally using river sources.

Our results support broader use of multiple sources for genetic augmentation during population rehabilitation and (re)introductions into former or new ranges. The need for such genetic management increases under changing climates. Frequency of catastrophic events such as prolonged heat waves, droughts, fires and floods are predicted to intensify, and so many isolated wildlife populations will be unable to adapt in situ or move to suitable habitats. In this case, they could benefit from assisted gene flow or translocation to a new habitat (Frankham et al., 2017), because more genetically diverse populations better survive climatic extremes (Reusch et al., 2005).

Given current rates of climate change and habitat loss and degradation, elevating population adaptive potential through genetic augmentation is essential for efficient conservation. For example, in 2020, over 185 species of plants and animals lost over half of their habitat in the Australian State of Victoria during catastrophic fires (DELWP 2020). These include 170 rare or threatened species (including Macquarie perch and other freshwater fish), whose remaining populations require urgent conservation management to prevent their extinction, including conservation breeding programmes and translocation to a more secure available habitat. Decision‐support tools for balancing the risk of harmful inbreeding under separate management and the risk of outbreeding depression after pooling remnant populations are available (Frankham et al., 2017), and cost‐effective genetic monitoring is now feasible for assessing success of different management strategies, as demonstrated by our study. The application of genetic management in conservation programmes is part of the growing adoption of evolutionary thinking in conservation management, and there is much room for greater uptake of the available resources for conservation managers. Leading organizations involved with conservation management are increasingly providing advice, tools and decision support for conservation managers, such as the International Union for the Conservation of Nature's recent development of the Conservation Genetics Specialist Group.

## 5 CONCLUSIONS

In agreement with a wealth of evolutionary theory about the genetic basis of fitness and adaptation, our study adds to growing evidence that using multiple compatible sources can result in substantial fitness benefits, especially for small and isolated populations (Frankham, 2015). Although the evolutionary mechanisms behind higher fitness of individuals of mixed ancestry might not always be readily distinguishable, using multiple sources during population reestablishment provides populations with higher effective population sizes and genetic diversity, thus greater resources for selection on phenotypic variation suited to the environment. Whereas a given source population might seem preferable due to higher fitness, it might not be always available for large‐scale translocations required to create initially viable populations, and other sources still contribute useful genetic variation through hybrids and backcrosses, as in the present case. Genetic monitoring will be beneficial to ensure backcrosses are produced, population sizes are increasing and effective size remains sufficient, with additional translocations performed if required (Weeks et al., 2015).

## Supporting information

Data S1

Data S2

Appendix S1

## Data Availability

Genotypic data for this study are available at Bridges research repository at https://doi.org/10.26180/5ea1736cc3553.
