# Supplementary material for: Using multiple sources during reintroduction of a locally extinct population benefits survival and reproduction of an endangered freshwater fish
Source: Evol Appl. 2020 Dec 15;14(4):950–64. doi: 10.1111/eva.13173 (PMC8061264; doi:10.1111/eva.13173)
Supplement: Supplementary file 3 — Appendix S1 [file EVA-14-950-s003.zip › eva_13173_AppenddixD_TR.docx]

**Appendix D. *Details of methods, results and data on growth, and calculations for the growth model for Ovens fish inferred to be stocked***

Methods

*Additional details of the growth models for Ovens fish inferred to be stocked*

Following Tonkin et al. (2017), we fitted a standard nonlinear regression model (Gompertz) of length Lx (mm) at age X (years),

$L_{x}=L_{\infty} e^{ e^{\left(-b\left(X-M\right)\right)}}$ where L*_x_* is the length at age *X*, L_∞_ is the asymptotic length, *b* is the instantaneous growth rate, and *M* is the inflection point of the curve,

to age and length data for 92 known-age Ovens individuals assigned broodstock parents. The initial parameters used in the Gompertz model were adopted from Tonkin et al. (2017). Age for fish inferred to be stocked was inferred as the difference between capture date and December 1^st^ of the year of mating of their parents.

Residuals calculated from the Gompertz model for the Ovens fish inferred to be stocked (reflecting the difference in their growth compared to the model average) were used as the response variable in linear mixed models testing for the effect of parental cross-type (genetic ancestry) and parental genetic dissimilarity or genetic diversity on fish growth. Parental cross-type and either parental dissimilarity or individual genetic diversity (PHt) were fitted as fixed effects, and year of stocking as a random effect, to account for interannual environmental variation likely to affect growth. The effect variables were scaled and centred using the scale function in R. The models were run using the lmer function in the R package lme4 (Bates et al., 2015) using the following script (input file is in Supporting Information 2):

### Growth model

# load some packages

library(readxl)

library(lme4)

library(lmerTest)

# load the data

growth_data <- read_xlsx("data/Supporting_Information_2_EVA_revised.xlsx",

sheet = "Growth_Model.csv")

# fit a non-linear growth model and extract residuals

mod_length_at_age <- nls(Length_caught ~ b1 * exp(-b2 * exp(b3 * Age_at_capture)),

data = growth_data,

start = list(b1 = 500, b2 = 0.5, b3 = 0.3),

algorithm = "port",

trace = TRUE,

control = list(maxiter = 1000, tol = 5e-4))

growth_data$Growth_Residuals <- resid(mod_length_at_age)

# rescale growth predictors

growth_data$PHt_std <- scale(growth_data$PHt)

growth_data$Genetic_distance_std <- scale(growth_data$Genetic_distance)

# fit mixed model of growth residuals against a set of genetic and

# cross-type predictors

vars <- c("PHt_std", "Crossing", "Genetic_distance_std",

"PHt_std + Crossing", "Genetic_distance_std + Crossing")

vars <- paste0(vars, " + ")

vars <- c("", vars)

growth_form <- paste0("Growth_Residuals ~ ", vars, "(1 | Release_year)")

growth_mod <- vector("list", length = length(growth_form))

for (i in seq_along(growth_form))

growth_mod[[i]] <- lmer(as.formula(growth_form[i]), data = growth_data, REML = FALSE)

# summarise the fitted growth models

growth_mod_summary <- lapply(growth_mod, summary)

# AIC comparison of fitted models

growth_mod_aic <- sapply(growth_mod, AIC)

# calculate r2 for each model

growth_mod_r2 <- lapply(growth_mod, MuMIn::r.squaredGLMM)

*Estimating ages of nonstocked (i.e. locally born and identified as translocated) Ovens fish*

Data for length and age of the Ovens fish inferred to be stocked enabled estimating parameters of the Gompertz model for age-specific fish growth in the Ovens River (the above script). We used these parameters to estimate ages for nonstocked Ovens fish from their lengths, using the following equation:

Age = LN(LN(Length/347.0091)/-1.94672)/-0.56074

The estimates of ages for nonstocked fish were used for analyses of local recruitment.

Results

*Different growth rates for Ovens fish inferred to be stocked of different ancestry*

The parental cross-type (genetic ancestry) was the only predictor included in the most parsimonious model (lowest AIC, Table D1). Models including cross type and offspring genetic diversity (PHt) or parental genetic dissimilarity both had ΔAIC ≤ 2 (Table D1). All three models explained approximately 0.07 of the variation in residual growth, with cross-type the only significant predictor of relative growth (Table D2). The offspring of Yarra x Yarra crosses were significantly bigger than those of Dartmouth x Dartmouth and Dartmouth x Yarra crosses of the same age (Table D2, Fig. 2b of the main manuscript). However, these results should be interpreted with caution, because Yarra x Yarra and Dartmouth x Yarra crosses were not performed in the same years (see Figs. 1a,b in the main manuscript): Yarra x Yarra crosses were performed until 2013/2014, and Dartmouth x Yarra—from 2014/2015). No significant relationship was detected between individual genetic diversity (PHt) of stocked Ovens individuals or genetic dissimilarity of their parents and their growth (Table D2; Fig 2a in the main manuscript).

*Distribution of ages of Ovens fish that were not assigned broodstock parents support local recruitment*

Ages estimated for the 210 sampled Ovens individuals for which broodstock parents were not assigned (Table D4, data in Supporting Information 1) showed that 92% of individuals were below the age of maturity (2 years old or younger) when sampled during monitoring.

**Table D1.** Results of model comparison of growth model with offspring genetic diversity, genetic dissimilarity between broodstock pairs and cross type. Year of stocking was included in all models as a random factor. Model comparison was based on Akaike’s Information Criterion (AIC). The lowest AIC value (most parsimonious model) is in boldface.

| **Included variables** | **AIC** |
| --- | --- |
| Individual genetic diversity | 779 |
| Intercept-only (null) model | 777.66 |
| Genetic dissimilarity | 777.02 |
| Genetic dissimilarity, cross-type | 776.66 |
| Individual genetic diversity, cross-type | 776.18 |
| Cross-type | **774.66** |

**Table D2**. Results of most parsimonious growth model: residual growth of stocked fish as a function of cross type, with year of stocking included as a random factor. Results are also shown for two models with ΔAIC ≤ 2: residual growth as a function of offspring genetic diversity and cross type, and residual growth as a function of genetic dissimilarity and cross type. R2m is a likelihood ratio-based pseudo R^2^ measure calculated with the R package MuMIn. Significant P-values are in bold.

|  | **Predictor** | **Coefficient** | **Standard error** | **Pr(>\|t\|)** | **R2m** |
| --- | --- | --- | --- | --- | --- |
| **(a)** | Parental cross-type |  |  |  | 0.07 |
|  | DxD | –3.67 | 2.87 | 0.203 |  |
|  | DxY | 2.34 | 3.68 | 0.526 |  |
|  | YxY | 12.07 | 4.63 | **0.011** |  |
| **(b)** | PHt | –1.51 | 2.19 | 0.152 | 0.08 |
|  | Parental cross-type |  |  |  |  |
|  | DxD | –5.11 | 3.54 | 0.491 |  |
|  | DxY | 4.24 | 4.58 | 0.357 |  |
|  | YxY | 14.67 | 5.97 | **0.016** |  |
| **(c)** | Parental dissimilarity | –0.12 | 3.24 | 0.440 | 0.07 |
|  | Parental cross-type |  |  |  |  |
|  | DxD | –3.82 | 4.92 | 0.971 |  |
|  | DxY | 2.54 | 6.49 | 0.697 |  |
|  | YxY | 12.32 | 8.45 | 0.148 |  |

**Table D3.** Minimum, mean and maximum length (mm) for fish of age classes from zero to 5, for 92 Ovens individuals inferred to be stocked.

| **Age class** | **Number of individuals** | **Min. length (mm)** | **Mean length (mm)** | **Max. length (mm)** |
| --- | --- | --- | --- | --- |
| 0 | 8 | 45 | 57.5 | 72 |
| 1 | 21 | 112 | 132.43 | 157 |
| 2 | 28 | 175 | 206.86 | 230 |
| 3 | 28 | 210 | 248.46 | 277 |
| 4 | 4 | 270 | 283.25 | 301 |
| 5 | 3 | 299 | 334.67 | 384 |

**Table D4.** Ages of the Ovens fish of different ancestry inferred to be stocked (parents assigned) and nonstocked (translocated and locally born) captured in each year of monitoring; six individuals sampled twice in different seasons (as inferred by identity analysis) are counted for each season and included in the total twice (a total of 204 nonstocked and 92 stocked individuals are unique). Ages for stocked fish are inferred based on date of mating of their parents. Ages for nonstocked fish are based on the Gompertz model parametrized using stocked fish of known age.

|  | **Year of monitoring** | **Genetic ancestry** | **Estimated age of fish during monitoring, years** | | | | |  |
| --- | --- | --- | --- | --- | --- | --- | --- | --- |
|  |  |  | **0** | **1** | **2** | **3** | **4+** | **Total** |
| **Nonstocked Ovens fish** | 2016 | Two-population | 4 | 8 |  |  |  | 12 |
|  |  | Dartmouth | 4 | 3 |  |  | 1 | 8 |
|  |  | Yarra | 6 | 6 | 3 | 0 |  | 15 |
|  |  | Total 2016 | 14 | 17 | 3 | 0 | 1 | 35 |
|  | 2017 | Two-population | 4 | 10 | 3 |  |  | 17 |
|  |  | Dartmouth |  |  | 1 |  | 1 | 2 |
|  |  | Yarra | 8 | 15 | 3 |  |  | 26 |
|  |  | Total 2017 | 12 | 25 | 7 |  | 1 | 46 |
|  | 2018 | Two-population | 2 | 14 | 15 | 5 | 2 | 38 |
|  |  | Dartmouth |  | 1 | 4 | 1 | 2 | 8 |
|  |  | Yarra | 1 | 56 | 20 | 4 |  | 81 |
|  |  | Total 2018 | 3 | 71 | 39 | 9 | 4 | 128 |
| **Total nonstocked Ovens** | | | **29** | **113** | **49** | **10** | **6** | **207** |
| **Stocked Ovens fish** | 2016 | Two-population | 3 | 13 |  |  |  | 16 |
|  |  | Dartmouth | 3 | 4 | 3 |  |  | 10 |
|  |  | Yarra |  |  | 6 | 1 |  | 7 |
|  |  | Total 2016 | 6 | 17 | 9 | 1 |  | 33 |
|  | 2017 | Two-population |  | 2 | 12 |  |  | 14 |
|  |  | Dartmouth |  |  | 2 | 1 |  | 3 |
|  |  | Yarra |  |  |  | 4 | 1 | 5 |
|  |  | Total 2017 |  | 2 | 14 | 5 | 1 | 22 |
|  | 2018 | Two-population |  |  | 3 | 13 |  | 16 |
|  |  | Dartmouth | 2 | 2 | 4 | 9 |  | 17 |
|  |  | Yarra |  |  |  |  | 7 | 7 |
|  |  | Total 2018 | 2 | 2 | 7 | 22 | 7 | 40 |
| **Total stocked Ovens** | | | **8** | **21** | **30** | **28** | **8** | **95** |
| **Total Ovens** | |  | **37** | **134** | **79** | **38** | **14** | **302** |

References

Bates, D., Maechler, M., Bolker, B., & Walker, S. (2015) Fitting Linear Mixed-Effects Models Using lme4. Journal of Statistical Software, 67(1), 1-48. doi: 10.18637/jss.v067.i01.

Tonkin, Z., Kearns, J., Lyon, J., Balcombe, S. R., King, A. J., & Bond, N. R. (2017) Regional‐scale extremes in river discharge and localised spawning stock abundance influence recruitment dynamics of a threatened freshwater fish. Ecohydrology, 10(6), e1842. doi: 10.1002/eco.1842.

Tonkin, Z., Lyon, J., Ramsey, D. S., Bond, N. R., Hackett, G., Krusic-Golub, K., ... Balcombe, S. R. (2014) Reservoir refilling enhances growth and recruitment of an endangered remnant riverine fish. Canadian Journal of Fisheries and Aquatic Sciences, 71(12), 1888–1899. doi: 10.1139/cjfas-2014-0081.
