# Supplementary material for: Using multiple sources during reintroduction of a locally extinct population benefits survival and reproduction of an endangered freshwater fish
Source: Evol Appl. 2020 Dec 15;14(4):950–64. doi: 10.1111/eva.13173 (PMC8061264; doi:10.1111/eva.13173)
Supplement: Supplementary file 3 — Appendix S1 [file EVA-14-950-s003.zip › eva_13173_AppendixA_TR.docx]

**Appendix A*. Details of stocking, translocation and monitoring of Macquarie perch in the Ovens River***

**Table A1.** Contribution of successful broodstock pairs to stocking to the Ovens River. A total of 63 Snobs Creek Hatchery broodstock pairs produced fingerlings that were stocked to the Ovens River. Each pair was used just once, but some individuals were paired more than once; a total of 108 broodstock fish were used. Breeding season—year of breeding/year of stocking; Cross-type—origin of mother x father; N larvae produced—hatchery estimate of the number of larvae produced by each pair; N fingerlings stocked to Ovens—estimated as the number of larvae produced by each pair divided by the total number of larvae produced that year by all broodstock fish, multiplied by the total number of fingerlings stocked that year to the Ovens. No genetic samples were collected for individuals marked with asterisk (*, red font). Twenty-four broodstock fish that were translocated to the Ovens River are marked with hash (^#^). MP_CBR70 (in blue font, marked with $) had a genotype identical to that of MP_CBR138, suggesting a potential sample mix-up.

| **Breeding season** | **Female ID** | **Male ID** | **Cross type** | **N larvae produced** | **N fingerlings stocked to Ovens** | |
| --- | --- | --- | --- | --- | --- | --- |
| 2010/2011 | MP_GBR28 | 000682D172* | Yarra x Yarra | 8,330 | 521 |  |
|  | MP_GBR30 | Unknown* | Yarra x NA | 150 | 9 |  |
|  | 000680D0B1* | MP_CBR82 | Dartmouth x Dartmouth | 1,450 | 91 |  |
|  | 000680B15C* | 0006809DED* | Dartmouth x Dartmouth | 24,800 | 1,552 |  |
|  | MP_CBR88 | MP_CBR86 | Dartmouth x Dartmouth | 23,700 | 1,483 |  |
|  | 000680B206* | MP_CBR87 | Dartmouth x Dartmouth | 1,450 | 91 |  |
|  | 000680C2FF* | 000682D172* | Yarra x Yarra | 2,450 | 153 |  |
| 2012/2013 | MP_GBR01 | MP_GBR07^#^ | Yarra x Yarra | 18,440 | 1,296 |  |
|  | MP_GBR02 | MP_GBR11 | Yarra x Yarra | 5,914 | 416 |  |
|  | MP_GBR03 | MP_GBR08 | Yarra x Yarra | 24,800 | 1,743 |  |
|  | MP_GBR04 | MP_GBR09^#^ | Yarra x Yarra | 14,400 | 1,012 |  |
|  | MP_GBR05 | MP_GBR10^#^ | Yarra x Yarra | 10,480 | 737 |  |
|  | MP_CBR03 | MP_CBR10 | Dartmouth x Dartmouth | 5,586 | 393 |  |
|  | MP_GBR22^#^ | 0006809bd7* | Yarra x Yarra | 10,300 | 724 |  |
| 2013/2014 | MP_GBR30 | MP_GBR51^#^ | Yarra x Yarra | 21,000 | 6,345 |  |
|  | MP_GBR05 | MP_GBR08 | Yarra x Yarra | 3,100 | 937 |  |
|  | MP_GBR62 | MP_GBR68^#^ | Yarra x Yarra | 12,000 | 3,626 |  |
|  | MP_GBR64 | MP_GBR70^#^ | Yarra x Yarra | 7,600 | 2,296 |  |
|  | MP_GBR65 | MP_GBR69^#^ | Yarra x Yarra | 4,275 | 1,292 |  |
|  | MP_GBR66 | 0006809CD5* | Yarra x Yarra | 10 | 3 |  |
|  | MP_GBR80 | MP_GBR79 | Yarra x Yarra | 350 | 106 |  |
|  | MP_CBR54 | MP_CBR69^#^ | Dartmouth x Dartmouth | 2,500 | 755 |  |
|  | MP_CBR55 | MP_CBR78 | Dartmouth x Dartmouth | 7,800 | 2,357 |  |
|  | MP_CBR56 | MP_CBR70^$^ | Dartmouth x Dartmouth | 6,800 | 2,055 |  |
|  | MP_CBR59 | MP_CBR71 | Dartmouth x Dartmouth | 7,150 | 2,160 |  |
|  | MP_CBR60 | MP_CBR73 | Dartmouth x Dartmouth | 7,550 | 2,281 |  |
|  | MP_CBR61 | MP_CBR72^#^ | Dartmouth x Dartmouth | 13,300 | 4,019 |  |
|  | MP_CBR62^#^ | MP_CBR74 | Dartmouth x Dartmouth | 13,000 | 3,928 |  |
|  | MP_CBR63^#^ | MP_CBR75^#^ | Dartmouth x Dartmouth | 7,000 | 2,115 |  |
|  | MP_CBR64 | MP_CBR77 | Dartmouth x Dartmouth | 1,400 | 423 |  |
|  | MP_CBR65 | x00067562E9* | Dartmouth x Dartmouth | 8,800 | 2,659 |  |
|  | MP_CBR67 | MP_CBR76 | Dartmouth x Dartmouth | 10,400 | 3,142 |  |
| 2014/2015 | MP_CBR91^#^ | MP_CBR99^#^ | Dartmouth x Dartmouth | 14,000 | 2,120 |  |
|  | MP_CBR91^#^ | MP_GBR14^#^ | Dartmouth x Yarra | 13,500 | 2,045 |  |
|  | MP_CBR92^#^ | MP_CBR100^#^ | Dartmouth x Dartmouth | 12,300 | 1,863 |  |
|  | MP_CBR92^#^ | MP_GBR09^#^ | Dartmouth x Yarra | 9,500 | 1,439 |  |
|  | MP_CBR93 | MP_CBR101^#^ | Dartmouth x Dartmouth | 12,650 | 1,916 |  |
|  | MP_CBR93 | MP_GBR67^#^ | Dartmouth x Yarra | 14,000 | 2,120 |  |
|  | MP_CBR94^#^ | MP_CBR102^#^ | Dartmouth x Dartmouth | 3,850 | 583 |  |
|  | MP_CBR94^#^ | MP_GBR60 | Dartmouth x Yarra | 3,500 | 530 |  |
|  | MP_CBR95^#^ | MP_CBR103 | Dartmouth x Dartmouth | 3,200 | 485 |  |
|  | MP_CBR95^#^ | MP_GBR53 | Dartmouth x Yarra | 3,300 | 500 |  |
| 2015/2016 | MP_CBR96 | MP_GBR79 | Dartmouth x Yarra | 3,400 | 1,741 |  |
|  | MP_CBR97^#^ | MP_GBR08 | Dartmouth x Yarra | 1,200 | 614 |  |
|  | MP_CBR98 | MP_CBR77 | Dartmouth x Dartmouth | 7,900 | 4,045 |  |
| 2016/2017 | MP_CBR139 | MP_CBR153 | Dartmouth x Dartmouth | 19,600 | 4,900 |  |
|  | MP_CBR140 | MP_CBR155 | Dartmouth x Dartmouth | 5,100 | 1,275 |  |
|  | MP_CBR141 | MP_CBR158 | Dartmouth x Dartmouth | 5,000 | 1,250 |  |
|  | MP_CBR142 | MP_CBR154 | Dartmouth x Dartmouth | 3,000 | 750 |  |
|  | MP_GBR30 | MP_CBR156 | Yarra x Dartmouth | 500 | 125 |  |
| 2017/2018 | MP_CBR1390 | MP_CBR1402 | Dartmouth x Dartmouth | 23,000 | 2,313 |  |
|  | MP_CBR1391 | MP_CBR1403 | Dartmouth x Dartmouth | 10,000 | 1,006 |  |
|  | MP_CBR1392 | MP_CBR1404 | Dartmouth x Dartmouth | 655 | 66 |  |
|  | MP_CBR1393 | MP_CBR1405 | Dartmouth x Dartmouth | 26,100 | 2,625 |  |
|  | MP_CBR1394 | MP_CBR1406 | Dartmouth x Dartmouth | 12,600 | 1,267 |  |
|  | MP_CBR1395 | MP_CBR1407 | Dartmouth x Dartmouth | 9,900 | 996 |  |
|  | MP_CBR1396 | MP_CBR1408 | Dartmouth x Dartmouth | 8,034 | 808 |  |
|  | MP_CBR1397 | MP_CBR1407 | Dartmouth x Dartmouth | 6,400 | 644 |  |
|  | MP_CBR1398 | MP_CBR1409 | Dartmouth x Dartmouth | 10,170 | 1,023 |  |
|  | MP_CBR1399 | MP_CBR1410 | Dartmouth x Dartmouth | 4,000 | 402 |  |
|  | MP_CBR1400 | MP_CBR1411 | Dartmouth x Dartmouth | 2,900 | 292 |  |
|  | MP_CBR1401 | MP_CBR103 | Dartmouth x Dartmouth | 29,700 | 2,987 |  |
|  | MP_GBR30 | MP_CBR1402 | Yarra x Dartmouth | 5,700 | 573 |  |

**Table A2.** Number of hatchery-produced fingerlings stocked into the Ovens River at each site (Fig. A1). The Ovens River was not stocked with fingerlings produced during the 2009/2010 and 2011/2012 breeding seasons.

| **Site name** | **Site coordinates** | **2011** | **2013** | **2014** | **2015** | **2016** | **2017** | **2018** | **N stocked per site** |
| --- | --- | --- | --- | --- | --- | --- | --- | --- | --- |
| Site 1 | -36.3628, 146.3477 |  |  | 6,500 |  |  |  |  | **6,500** |
| Site 2 | -36.3795, 146.3664 |  |  |  | 2,720 | 700 | 2,000 |  | **5,420** |
| Site 5 | -36.3795, 146.3664 |  |  | 6,500 | 2,720 |  |  |  | **9,220** |
| Site 6 | -36.4211, 146.4897 |  |  |  |  |  |  | 5,000 | **5,000** |
| Site 7 | -36.4654, 146.5389 |  |  |  |  |  | 2,300 | 5,000 | **7,300** |
| Site 8 | -36.4700, 146.5477 |  |  |  |  |  | 2,000 | 5,000 | **7,000** |
| Site 9 | -36.4736, 146.5463 |  |  |  |  |  | 2,000 |  | **2,000** |
| Site 10 | -36.5026, 146.6040 | 1,400 | 3,160 | 10,500 | 2,720 | 1,900 |  |  | **19,680** |
| Site 13 | -36.5282, 146.6627 | 2,500 | 3,160 | 10,500 | 2,720 | 1,900 |  |  | **20,780** |
| Site 14 | -36.5417, 146.6723 |  |  | 6,500 | 2,720 | 1,900 |  |  | **11,120** |
|  | **N stocked per year** | **3,900** | **6,320** | **40,500** | **13,600** | **6,400** | **8,300** | **15,000** | **94,020** |

**Table A3**. Number of individuals translocated to the Ovens River per site (see map in Fig. A1). Retired broodstock fish (Supporting Information S1) were translocated in 2016.

| **Site name** | **Site coordinates** | **2014** | **2015** | **2016** | **2017** | **N translocated per site** |
| --- | --- | --- | --- | --- | --- | --- |
| Site 3 | -36.3842, 146.3904 |  |  |  | 175 | **175** |
| Site 4 | -36.3832, 146.3912 |  |  | 58 |  | **58** |
| Site 5 | -36.3795, 146.3664 | 114 | 190 | 122 | 160 | **586** |
| Site 6 | -36.4211, 146.4897 |  |  |  | 140 | **140** |
| Site 11 | -36.5157, 146.6237 |  |  | 102 |  | **102** |
| Site 12 | -36.5261, 146.6384 |  |  | 30 | 17 | **47** |
| Site 14 | -36.5417, 146.6723 | 112 | 300 | 101 | 180 | **693** |
|  | **N translocated per year** | **226** | **490** | **413** | **672** | **1,801** |

**Table A4.** Number of individuals genetically sampled during Ovens River monitoring per site (Fig. A1). Sites 31 and 32 are at Buffalo River, a tributary of the Ovens River, to which fish could naturally disperse. Surveys occurred during periods of low river flow to minimise variations in electrofishing efficiency. The intensity of surveys differed among years. In 2016 and 2017 surveys were conducted at 14 sites spanning ~100 km of the Ovens River; in 2018—at 32 sites spanning ~180 km of the Ovens River and five sites in the Buffalo River.

| **Site name** | **Site coordinates** | **2016** | **2017** | **2018** | **N sampled per site** |
| --- | --- | --- | --- | --- | --- |
| Site 1 | -36.3628, 146.3477 |  |  | 4 | **4** |
| Site 2 | -36.3795, 146.3664 | 3 |  | 4 | **7** |
| Site 3 | -36.3842, 146.3904 |  |  | 5 | **5** |
| Site 5 | -36.3795, 146.3664 | 9 | 11 | 5 | **25** |
| Site 7 | -36.4654, 146.5389 |  |  | 7 | **7** |
| Site 10 | -36.5026, 146.6040 |  | 21 | 5 | **26** |
| Site 12 | -36.5261, 146.6384 |  | 18 | 25 | **43** |
| Site 13 | -36.5282, 146.6627 | 8 |  | 37 | **45** |
| Site 14 | -36.5417, 146.6723 | 25 | 17 | 15 | **57** |
| Site 16 | -36.3565, 146.3357 | 1 |  |  | **1** |
| Site 17 | -36.3554, 146.3417 | 1 |  |  | **1** |
| Site 18 | -36.3711, 146.3587 | 1 |  |  | **1** |
| Site 19 | -36.3639, 146.3502 | 6 |  | 4 | **10** |
| Site 20 | -36.5082, 146.6083 | 6 |  |  | **6** |
| Site 21 | -36.5061, 146.6047 | 4 |  |  | **4** |
| Site 22 | -36.5258, 146.6379 | 4 |  |  | **4** |
| Site 23 | -36.4014, 146.4331 |  |  | 2 | **2** |
| Site 24 | -36.4399, 146.5221 |  |  | 11 | **11** |
| Site 25 | -36.2527, 146.4897 |  |  | 1 | **1** |
| Site 26 | -36.6018, 146.7628 |  |  | 1 | **1** |
| Site 27 | -36.5462, 146.6779 |  |  | 13 | **13** |
| Site 28 | -36.5783, 146.7296 |  |  | 3 | **3** |
| Site 29 | -36.6169, 146.8047 |  |  | 2 | **2** |
| Site 30 | -36.5518, 146.6889 |  |  | 9 | **9** |
| Site 31 | -36.6583, 146.6921 |  |  | 1 | **1** |
| Site 32 | -36.5665, 146.6908 |  |  | 13 | **13** |
|  | **N sampled per year** | **68** | **67** | **167** | **302** |


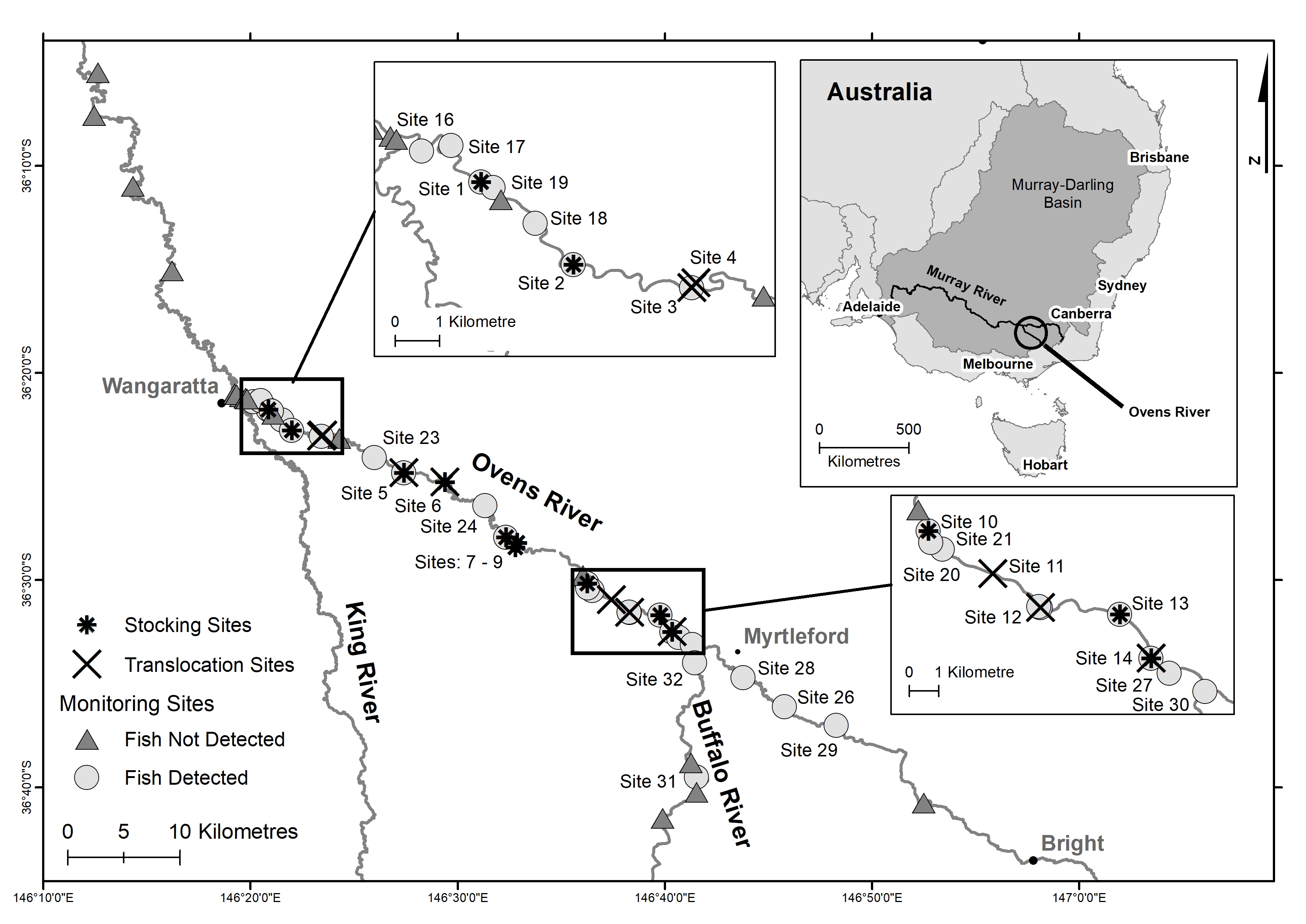


**Figure A1.** Map of sites of stocking (2010-2017, asterisk), translocation (2014-2017, crosses) and monitoring (2016-2017, circles- sampling sites, triangles- sites with no fish detected) of the Macquarie perch in the Ovens and Buffalo Rivers. Sampling was done at, or very near, all translocation and stocking sites in the last year of monitoring (Tables A2-A4).


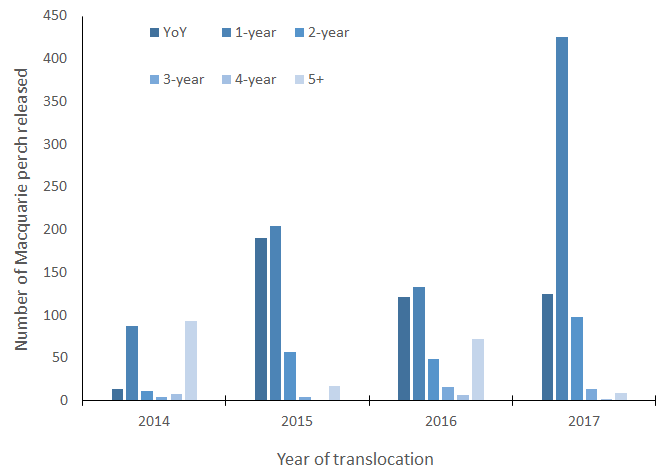


**Figure A2**. Distributions of age classes from young-of-year (YoY, darkest blue) to five years and older (5+, lightest blue) for individuals translocated to the Ovens River from Lake Dartmouth between 2014 and 2017. Age estimates are based on Gompertz growth model by Tonkin et al. (2017) for Dartmouth:

Age = LN(LN(Length/421)/-1.83718)/-0.37764

Red arrows indicate age classes that were used only for identity analyses (i.e. individuals that were <2 years old by December 2017 and thus assumed to be too young to be parents of the fish captured during Ovens monitoring).
