# Supplementary material for: Using multiple sources during reintroduction of a locally extinct population benefits survival and reproduction of an endangered freshwater fish
Source: Evol Appl. 2020 Dec 15;14(4):950–64. doi: 10.1111/eva.13173 (PMC8061264; doi:10.1111/eva.13173)
Supplement: Supplementary file 3 — Appendix S1 [file EVA-14-950-s003.zip › eva_13173_AppendixB_TR.docx]

**Appendix B: *Details of parentage, identity, sibship and hybrid class assignment analyses***

*Parentage analysis*

Methods

To select an appropriate genotyping error rate for parentage analysis, we ran a calibration parentage analysis in CERVUS3, which included genotypes of 30 hatchery-produced fingerlings from the 2012 breeding season and genotypes of five pairs of known parents of these fingerlings, identified previously using microsatellites (Pavlova et al., unpublished data). The calibration analysis used a genotyping dataset for 2,704 loci scored in 301 individuals. It included 30 hatchery fingerlings and 135 Ovens fish as potential offspring, and 136 candidate parents (broodstock fish, including 76 that contributed fingerlings to the Ovens stocking; see Supporting Information 1 for the list of individuals included). With error rate of 0.0001, all known parent–offspring relationships were correctly detected and all false parent–offspring relationships were correctly rejected.

For the final parentage analysis, we used a genotyping dataset for 1,204 loci scored in 1,679 individuals with <25% missing data (see Supporting Information 1 for the list of individuals included; 41 Dartmouth fish translocated to the Ovens were excluded by this filter, of which 29 were born in 2014 or earlier, and thus could have been parents of the young Ovens fish that were not assigned broodstock parents). The 302 Ovens fish were included as potential offspring and all broodstock and translocated fish of breeding age (>2 years old) as potential parents (see Supporting Information 1 for the list of individuals included). Only relationships where offspring were assigned both parents were considered, because some single-parent assignments in our test data resulted in parent–offspring pairs that were impossible given known translocation/stocking history and inferred ages of fish. To avoid incorrectly inferring parents, we cross-checked the proportion of loci mismatching between each offspring and both inferred parents (calculated by CERVUS3) and rejected parent–offspring pairs with more than 0.40% loci mismatching, based on the empirical rate for the 30 known parent–offspring groups.

Results

A total of 92 fish sampled in the Ovens River were assigned both a mother and a father consistent with known broodstock pairs (Table B1); these 92 fish were inferred to be stocked. A total of 20 different mothers and 22 fathers were identified. Three broodstock pairs from 2013 and 2014 breeding seasons produced more than 40% of all stocked offspring detected in the Ovens: two Dartmouth x Yarra crosses and one Yarra x Yarra:

Dartmouth x Yarra: MP_CBR91 and MP_GBR14, 13 offspring;

Dartmouth x Yarra: MP_CBR92 and MP_GBR09, 16 offspring;

Yarra x Yarra: MP_GBR62 and MP_GBR68, 10 offspring.

Two Dartmouth x Yarra crosses produced 65% of the stocked individuals detected in the most successful breeding season of 2014:

MP_CBR91 and MP_GBR14, 7 offspring;

MP_CBR92 and MP_GBR09, 13 offspring.

A single Dartmouth female MP_CBR92 engaged in two broodstock crosses was assigned as a mother of 21 stocked offspring, including five and 16 offspring of two crosses, with MP_CBR100 (Dartmouth) and with MP_GBR09 (Yarra), respectively (Table B1).

In addition to detecting stocked offspring of hatchery broodstock fish, four non-stocked Ovens fish were assigned both parents among translocated Dartmouth fish, indicating their local recruitment:

MP_OV048, captured in 2016, inferred to be an offspring of MP_Tr2014_027 and MP_Tr2014_043, translocated in 2014;

MP_OV_2018_222, captured in 2018, inferred to be an offspring of MP_CBR164 and MP_Tr2014_036, translocated in 2014;

MP_OV009, captured in 2016, and MP_OV_2018_238, captured in 2018, inferred to be the offspring of MP_Tr2014_062 and MP_Tr2014_011, translocated in 2014.

One confident (at 95%) parentage assignment was considered incorrect and excluded from further analyses: MP_OV061 (not in table B1) was assigned MP_GBR28 and MP_GBR10 as candidate parents by CERVUS3 analysis (offspring–mother and offspring–father pairs had ≤0.18% loci mismatch). The female MP_GBR28 was used in breeding crosses in 2010 but no genetic sample of the male used in the crossing was collected; she was not released to the Ovens. The potential offspring, MP_OV061, was only 68 mm long when collected during monitoring in 2016 (<1 year old) thus couldn’t have been born in 2010 when the potential parents were crossed. The assigned father, MP_GBR10, was released in the Ovens River in March 2016; however, MP_OV061 was sampled before this release.

*Identity analysis*

Results

In 17 instances, a pair of samples was inferred to have been derived from the same individual, in each case <0.5% of genotyped loci mismatched between two samples (Table B2). Six pairs corresponded to three stocked and three nonstocked Ovens individuals captured twice during monitoring. Four individuals collected during monitoring were identified as translocated Dartmouth fish (MP_OV011, MP_OV056, MP_OV_2018_160, MP_OV_2018_310; Table B2).

Seven instances of likely mislabelling error were also detected, where the same individual was genotyped twice under different IDs. Five involved fish translocated from Dartmouth in 2014 and 2015, suggesting that we were missing five potential parents of the Dartmouth origin, in addition to 41 that had too much missing data. One involved MP_GBR23 and MP_GBR25, broodstock fish from Yarra that did not contribute to the Ovens stocking. Another involved MP_CBR70, a broodstock male that contributed to the Ovens stocking, and male MP_CBR138 that did not, both from Dartmouth. The latter result suggests that the broodstock pair MP_CBR56/MP_CBR70 that did contribute to the Ovens stocking (Table A1) might not have been represented in our analyses. Nevertheless, female MP_CBR56 does not have any offspring assigned to it in COLONY2 or CERVUS3, suggesting this error is non-consequential for our conclusions. Given that a total of 2,173 fish were included in identity analysis, mislabelling errors represent 0.3%.

*Sibship analysis*

Results

Colony2 detected 40 full-sib groups (Table B3; Supporting Information 1). Of these, 16 groups included offspring of broodstock parents, one (group 16) included offspring of two translocated fish, and 23 groups included presumably locally born fish (offspring of either two stocked parents, one stocked and one translocated parents or other combinations involving one or both parents with missing genotypes). Groups inferred to be locally born included up to 11 full-sibs (group 7).

Full-sib group 19 included a combination of three individuals that was not realistic. Two of these, MP_OV043 and MP_OV_2018_225 were found to be identical by identity analysis, inferred from their length to have been born in 2012 and <2014, respectively, assigned broodstock parents by parentage analysis (MP_GBR04/MP_GBR09 bred in 2012), and thus inferred to be stocked (Supporting Information 1). The third individual assigned by Colony2 as their sibling, MP_OV_2018_178, was born in 2016 based on its length, whereas broodstock female MP_GBR04 was not released to the Ovens; hence MP_GBR04 could not have been the mother of MP_OV_2018_178. Thus, we infer MP_OV_2018_178 to locally-born to unknown parents.

*Hybrid class assignment*

Methods

NEWHYBRIDS analysis was performed to assign locally-born Ovens individuals (i.e. those not assigned broodstock parents and not inferred to be translocated by identity analysis) to Yarra or Dartmouth populations or one of four hybrid classes (F_1_, F_2_, backcross to Dartmouth, backcross to Yarra) with probability of P>0.5. A total of 338 individuals were included in the analysis (see Supporting Information 1 for the list): all 296 Ovens individuals and 25 Dartmouth and 17 Yarra broodstock fish that were assigned as parents of Ovens fish. The function *gl.nhybirds* of dartR R package was used to subsample 370 loci from the set of 407 used for STRUCTURE (i.e. loci most differentiated between Dartmouth and Yarra; *F*_ST_≥0.02) based on the AvgPIC: the average of the polymorphism information content (PIC) of the Reference and SNP alleles). The program would not run with more loci. Known Dartmouth fish, inferred (based on parentage analysis) stocked DxD offspring and translocated fish from Dartmouth were assigned to Dartmouth population (option z0), known Yarra fish and inferred stocked YxY offspring- to Yarra population (z1) and inferred stocked DxY- to F_1_ hybrids (option z2). Jeffrey’s prior for *π* and *θ* was used*.* Twenty replicate runs of 10,000 burnin and 10,000 sweeps each were performed and results averaged.

Results

NEWHYBRIDS correctly assigned all individuals pre-assigned using z option to Dartmouth (with P>0.7), Yarra (P>0.7) or F_1_ (P>0.59) (Supporting Information S1). NEWHYBRIDS assignments of 200 locally born Ovens fish (i.e. not assigned broodstock parents or inferred to be translocated) were generally consistent with STRUCTURE-based assignments: NEWHYBRIDS assigned 15 fish to Dartmouth (including 4 locally born offspring of translocated parent pair), 118 to Yarra and 67 to two-population hybrids (Supporting Information S1). The two exceptions were MP_OV_2018_305, assigned to Dartmouth by NEWHYBRIDS but two-population ancestry by STRUCTURE, and MP_OV_2018_181, assigned to F_1_ by NEWHYBRIDS but to Yarra by STRUCTURE; the latter was a member of sibship group 35, with its sibling assigned to Yarra, meaning it was more likely to be a Yarra genotype, consistent with the STRUCTURE result.

With P>0.5, 63 of 67 locally-born two-population hybrids were assigned to F_1_s, and two (MP_OV_2018_284 and MP_OV_2018_303), to backcrosses to Yarra. The remaining two fish did not belong to any ancestry class with P>0.5. Of these, MP_OV128 was assigned to F_2_ (P= 0.480) and F_1_ (P= 0.438), but its three full-sibs (from sibship group 1) were assigned to F_1_, making F_1_ the more likely designation for MP_OV128. The remaining fish, MP_OV_2018_237, was assigned to F_2_ (P=0.386), backcross to Yarra (P=0.384) and F_1_ (P=0.228), but it was inferred to be born in 2015, too early to be a plausible F_2_, as follows.

Given that some Macquarie perch of each sex can reach maturity by 3 years of age, fish of Yarra and Dartmouth ancestry stocked in 2011 could have interbred in the Ovens from 2013/14, and produce F_1_ and backcrosses from 2016/17, while DxY offspring stocked in 2015 could have produced F_1_s and backcrosses from 2017/18. The two inferred backcrosses were inferred to be born in 2016, and thus are the result of local reproduction by locally born DxY and (locally born or stocked) Yarra parents, which supports successful reproduction of F_1_ fish in the Ovens. Meanwhile, the two fish of uncertain ancestry class were born in 2015, before local F_2_ or backcrosses could have been produced, and thus must be F_1_ hybrids.

**Table B1.** Results of parentage analyses in CERVUS3 and COLONY2 (the list of individuals with assigned parents is in Supporting Information 1). All but one mother-father-offspring triads were assigned by both programs with a probability of 1. The exception was a pair MP_GBR04/MP_GBR09, for which three offspring were confidently assigned only by CERVUS3; of these offspring MP_OV043 and MP_OV_2018_225 were shown to be same individual by identity analysis and MP_OV_2018_178 (marked with asterisk) could not have been an offspring of MP_GBR04 based on its inferred age, as MP_GBR04 was not released in the Ovens; see text above). YxY: Yarra x Yarra crosses; DxY: Dartmouth x Yarra crosses; DxD: Dartmouth x Dartmouth crosses. Offspring identified as the same individual using identity analysis are highlighted in red italics. MP_OV_2018_282 and MP_OV_2018_277 (blue font) were sampled in Buffalo River.

| **Breeding season (November)** | **Candidate mother ID** | **Candidate father ID** | **Type of cross** | **Year of monitoring** | **Offspring ID** | **Total number of offspring** |
| --- | --- | --- | --- | --- | --- | --- |
| **2012** | MP_GBR01 | MP_GBR07 | YxY | 2018 | MP_OV_2018_163 | 1 |
|  | MP_GBR03 | MP_GBR08 | YxY | 2018 | MP_OV_2018_268, MP_OV_2018_290 | 2 |
|  | MP_GBR04 | MP_GBR09 | YxY | 2016  2018 | *MP_OV043*  *MP_OV_2018_225,* MP_OV_2018_178* | 1 |
|  | MP_GBR05 | MP_GBR10 | YxY | 2017 | MP_OV122 | 1 |
| **2013** | MP_CBR60 | MP_CBR73 | DxD | 2016 | MP_OV035 | 1 |
|  | MP_CBR67 | MP_CBR76 | DxD | 2016  2017 | MP_OV008, MP_OV019  MP_OV121 | 3 |
|  | MP_GBR62 | MP_GBR68 | YxY | 2016  2017  2018 | MP_OV021, MP_OV023, MP_OV025, MP_OV026, MP_OV033, MP_OV037  MP_OV081, MP_OV084, MP_OV103  MP_OV_2018_185 | 10 |
|  | MP_GBR64 | MP_GBR70 | YxY | 2017  2018 | MP_OV083  MP_OV_2018_201, MP_OV_2018_224 | 3 |
| **2014** | MP_CBR91 | MP_CBR99 | DxD | 2018 | MP_OV_2018_207, MP_OV_2018_315, MP_OV_2018_316 | 3 |
|  | MP_CBR91 | MP_GBR14 | DxY | 2016  2017  2018 | MP_OV004, MP_OV007, MP_OV017, MP_OV032  MP_OV068, MP_OV079, *MP_OV082*  MP_OV_2018_166, MP_OV_2018_186, MP_OV_2018_204, *MP_OV_2018_226*, MP_OV_2018_227, MP_OV_2018_246, MP_OV_2018_282 | 13 |
|  | MP_CBR92 | MP_CBR100 | DxD | 2016  2017  2018 | MP_OV016  MP_OV107  MP_OV_2018_161, MP_OV_2018_167, MP_OV_2018_252 | 5 |
|  | MP_CBR92 | MP_GBR09 | DxY | 2016  2017  2018 | MP_OV003, MP_OV006, MP_OV014, MP_OV020, MP_OV031  MP_OV071, MP_OV072, MP_OV073, MP_OV074, MP_OV080, MP_OV085, MP_OV092, MP_OV093  MP_OV_2018_168, MP_OV_2018_223, MP_OV_2018_242 | 16 |
|  | MP_CBR93 | MP_CBR101 | DxD | 2016  2017  2018 | MP_OV001, MP_OV010, MP_OV013  MP_OV113  *MP_OV_2018_169, MP_OV_2018_170*, MP_OV_2018_200, MP_OV_2018_257 | 7 |
|  | MP_CBR93 | MP_GBR67 | DxY | 2018 | MP_OV_2018_260, MP_OV_2018_270 | 2 |
|  | MP_CBR94 | MP_GBR60 | DxY | 2016  2017  2018 | MP_OV027, MP_OV047  MP_OV133  MP_OV_2018_311 | 4 |
|  | MP_CBR95 | MP_CBR103 | DxD | 2018 | MP_OV_2018_206 | 1 |
|  | MP_CBR95 | MP_GBR53 | DxY | 2016  2017 | MP_OV015  MP_OV109 | 2 |
| **2015** | MP_CBR96 | MP_GBR79 | DxY | 2016  2017  2018 | MP_OV049, MP_OV055  MP_OV086  MP_OV_2018_277 | 4 |
|  | MP_CBR97 | MP_GBR08 | DxY | 2016  2017  2018 | MP_OV051  MP_OV088  MP_OV_2018_157, MP_OV_2018_235 | 4 |
|  | MP_CBR98 | MP_CBR77 | DxD | 2016  2018 | MP_OV005, MP_OV063, MP_OV065  MP_OV_2018_228, MP_OV_2018_231 | 5 |
| **2016** | MP_CBR139 | MP_CBR153 | DxD | 2018 | MP_OV_2018_156 | 1 |
|  | MP_CBR141 | MP_CBR158 | DxD | 2018 | MP_OV_2018_317 | 1 |
| **2017** | MP_CBR1393 | MP_CBR1405 | DxD | 2018 | MP_OV_2018_155 | 1 |
|  | MP_CBR1400 | MP_CBR1411 | DxD | 2018 | MP_OV_2018_251 | 1 |

**Table B2**: Macquarie perch samples identified as the same individual during identity analysis in CERVUS3.

| **Sample 1** | **Sample 2** | **Proportion of loci mis-matching** | **Inference** |
| --- | --- | --- | --- |
| MP_OV_2018_169 | MP_OV_2018_170 | 0.0037 | Stocked, sampled in Ovens twice in 2018 |
| MP_OV_2018_225 | MP_OV043 | 0.0009 | Stocked, sampled in Ovens in 2016 and 2018 |
| MP_OV_2018_226 | MP_OV082 | 0.0037 | Stocked, sampled in Ovens in 2017 and 2018 |
| MP_OV_2018_153 | MP_OV077 | 0.0019 | Sampled in Ovens in 2017 and 2018 |
| MP_OV_2018_258 | MP_OV129 | 0.0019 | Sampled in Ovens in 2017 and 2018 |
| MP_OV034 | MP_OV106 | 0.0038 | Sampled in Ovens in 2016 and 2017 |
| MP_OV011 | MP_Tr2015_636 | 0.0046 | Sampled in Ovens in 2016/translocated from Dartmouth as YOY in 2015 |
| MP_OV056 | MP_C209 | 0.0018 | Sampled in Ovens in 2016/translocated from Dartmouth as adult in 2014 |
| MP_OV_2018_160 | MP_Tr2015_408 | 0.0027 | Sampled in Ovens in 2018/translocated from Dartmouth as 1YO in 2015 |
| MP_OV_2018_310 | MP_Tr2015_263 | 0.0009 | Sampled in Ovens in 2018/translocated from Dartmouth as 1YO in 2015 |
| MP_CBR138 | MP_CBR70 | 0 | Sampling or labelling error involving broodstock male MP_CBR70 |
| MP_GBR23 | MP_GBR25 | 0.0018 | Sampling or labelling error for fish translocated from Yarra |
| MP_Tr2014_104 | MP_Tr2014_105 | 0 | Sampling or labelling error for fish translocated from Dartmouth |
| MP_Tr2015_449 | MP_Tr2015_594 | 0.0009 | Sampling or labelling error for fish translocated from Dartmouth |
| MP_Tr2015_460 | MP_Tr2015_532 | 0.0009 | Sampling or labelling error for fish translocated from Dartmouth |
| MP_Tr2015_507 | MP_Tr2015_508 | 0.0028 | Sampling or labelling error for fish translocated from Dartmouth |
| MP_Tr2015_468 | MP_Tr2015_610 | 0.0046 | Sampling or labelling error for fish translocated from Dartmouth |

**Table B3** Full sibship assignments from Colony2, with added information on inferred parents and sibling chosen to represent its full-sibship group in Structure analysis. Individuals found to be identical using identity analysis are highlighted in red italics. Prob(Inc.) and Prob(Exc.) are the inclusive and exclusive probability of each family, respectively. Mother and father are either inferred broodstock parents or inferred unsampled parents (from output file BestCluster, starting with # or *). Group 19 included an unlikely combination of individuals (see above). Of two broodstock fish identified as fathers in group 6 and group 28 (highlighted in blue), male MP_CBR69 was released to the Ovens, and MP_CBR10 was not.

| **Group ID** | **Group size** | **Prob(Inc.)** | **Prob(Exc.)** | **Mother** | **Father** | **Sibling included in STRUCTURE** | **All siblings in the group** |
| --- | --- | --- | --- | --- | --- | --- | --- |
| Group 1 | 4 | 1 | 1 | #1 | *1 | MP_OV050 | MP_OV054, MP_OV050, MP_OV128, MP_OV_2018_205 |
| Group 2 | 16 | 0.9999 | 0.9999 | MP_CBR92 | MP_GBR09 | MP_OV003 | MP_OV014, MP_OV020, MP_OV003, MP_OV006, MP_OV031, MP_OV072, MP_OV080, MP_OV073, MP_OV074, MP_OV092, MP_OV085, MP_OV093, MP_OV071, MP_OV_2018_223, MP_OV_2018_242, MP_OV_2018_168 |
| Group 3 | 4 | 1 | 1 | MP_CBR96 | MP_GBR79 | MP_OV055 | MP_OV055, MP_OV049, MP_OV086, MP_OV_2018_277 |
| Group 4 | 5 | 0.9999 | 0.9999 | MP_CBR98 | MP_CBR77 | MP_OV063 | MP_OV063, MP_OV065, MP_OV005, MP_OV_2018_231, MP_OV_2018_228 |
| Group 5 | 2 | 1 | 1 | MP_CBR95 | MP_GBR53 | MP_OV109 | MP_OV015, MP_OV109 |
| Group 6 | 2 | 0.999 | 0.999 | #3 | MP_CBR69 | MP_OV_2018_278 | MP_OV056, MP_OV_2018_278 |
| Group 7 | 11 | 0.9999 | 0.9999 | #4 | *3 | MP_OV060 | MP_OV064, MP_OV060, MP_OV132, MP_OV069, MP_OV134, MP_OV135, MP_OV_2018_214, MP_OV_2018_202, MP_OV_2018_179, MP_OV_2018_211, MP_OV_2018_165 |
| Group 8 | 5 | 0.9999 | 0.9999 | MP_CBR92 | MP_CBR100 | MP_OV_2018_167 | MP_OV016, MP_OV107, MP_OV_2018_252, MP_OV_2018_167, MP_OV_2018_161 |
| Group 9 | 14 | 0.9999 | 0.9999 | MP_CBR91 | MP_GBR14 | MP_OV017 | MP_OV017, MP_OV068, MP_OV032, MP_OV004, MP_OV007, *MP_OV082*, MP_OV079, *MP_OV_2018_226*, MP_OV_2018_246, MP_OV_2018_227, MP_OV_2018_166, MP_OV_2018_186, MP_OV_2018_204, MP_OV_2018_282 |
| Group 10 | 8 | 0.9999 | 0.9999 | #5 | *5 | MP_OV059 | MP_OV058, MP_OV059, MP_OV044, MP_OV045, MP_OV046, MP_OV_2018_307, MP_OV_2018_164, MP_OV_2018_189 |
| Group 11 | 3 | 0.9999 | 0.9999 | MP_CBR67 | MP_CBR76 | MP_OV008 | MP_OV019, MP_OV008, MP_OV121 |
| Group 12 | 4 | 1 | 1 | #9 | *9 | MP_OV_2018_190 | MP_OV061, MP_OV127, MP_OV_2018_261, MP_OV_2018_190 |
| Group 13 | 8 | 0.9999 | 0.9999 | MP_CBR93 | MP_CBR101 | MP_OV_2018_257 | MP_OV013, MP_OV001, MP_OV010, MP_OV113, MP_OV_2018_257, MP_OV_2018_200, *MP_OV_2018_169*, *MP_OV_2018_170* |
| Group 14 | 10 | 0.9999 | 0.9999 | MP_GBR62 | MP_GBR68 | MP_OV021 | MP_OV033, MP_OV025, MP_OV026, MP_OV037, MP_OV021, MP_OV023, MP_OV081, MP_OV084, MP_OV103, MP_OV_2018_185 |
| Group 15 | 3 | 1 | 0.9995 | #10 | *4 | MP_OV041 | MP_OV041, MP_OV038, MP_OV_2018_233 |
| Group 16 | 2 | 1 | 0.9956 | MP_Tr2014_062 | MP_Tr2014_011 | MP_OV009 | MP_OV009, MP_OV_2018_238 |
| Group 17 | 2 | 1 | 1 | #9 | *14 | MP_OV034 | MP_OV034, MP_OV106 |
| Group 18 | 3 | 1 | 0.9999 | #13 | *13 | MP_OV_2018_314 | MP_OV002, MP_OV_2018_240, MP_OV_2018_314 |
| Group 19 | 3 | 1 | 1 | MP_GBR04 | MP_GBR09 | MP_OV_2018_178 | *MP_OV043*, *MP_OV_2018_225*, MP_OV_2018_178 |
| Group 20 | 4 | 1 | 1 | MP_CBR97 | MP_GBR08 | MP_OV_2018_157 | MP_OV051, MP_OV088, MP_OV_2018_235, MP_OV_2018_157 |
| Group 21 | 4 | 1 | 1 | MP_CBR94 | MP_GBR60 | MP_OV047 | MP_OV027, MP_OV047, MP_OV133, MP_OV_2018_311 |
| Group 22 | 3 | 0.9999 | 0.9999 | #15 | *18 | MP_OV029 | MP_OV028, MP_OV029, MP_OV030 |
| Group 23 | 2 | 1 | 1 | #20 | *20 | MP_OV_2018_258 | MP_OV129, MP_OV_2018_258 |
| Group 24 | 2 | 0.9844 | 0.9844 | #23 | *4 | MP_OV130 | MP_OV130, MP_OV131 |
| Group 25 | 2 | 0.9985 | 0.9985 | #19 | *25 | MP_OV110 | MP_OV114, MP_OV110 |
| Group 26 | 3 | 0.9988 | 0.9988 | #1 | *2 | MP_OV_2018_184 | MP_OV123, MP_OV_2018_184, MP_OV_2018_171 |
| Group 27 | 3 | 1 | 1 | MP_GBR64 | MP_GBR70 | MP_OV_2018_201 | MP_OV083, MP_OV_2018_224, MP_OV_2018_201 |
| Group 28 | 2 | 1 | 1 | #26 | MP_CBR10 | MP_OV100 | MP_OV099, MP_OV100 |
| Group 29 | 2 | 1 | 1 | #28 | *29 | MP_OV_2018_153 | MP_OV077, MP_OV_2018_153 |
| Group 30 | 2 | 1 | 1 | #2 | *38 | MP_OV_2018_313 | MP_OV_2018_267, MP_OV_2018_313 |
| Group 31 | 2 | 1 | 1 | MP_CBR93 | MP_GBR67 | MP_OV_2018_260 | MP_OV_2018_260, MP_OV_2018_270 |
| Group 32 | 2 | 0.9999 | 0.9999 | #16 | *10 | MP_OV_2018_304 | MP_OV_2018_175, MP_OV_2018_304 |
| Group 33 | 4 | 0.9999 | 0.9999 | #8 | *45 | MP_OV_2018_292 | MP_OV_2018_191, MP_OV_2018_300, MP_OV_2018_273, MP_OV_2018_292 |
| Group 34 | 2 | 0.9902 | 0.9902 | MP_GBR03 | MP_GBR08 | MP_OV_2018_268 | MP_OV_2018_268, MP_OV_2018_290 |
| Group 35 | 2 | 1 | 0.9999 | #45 | *27 | MP_OV_2018_276 | MP_OV_2018_276, MP_OV_2018_181 |
| Group 36 | 3 | 0.9999 | 0.9999 | #46 | *39 | MP_OV_2018_220 | MP_OV_2018_199, MP_OV_2018_209, MP_OV_2018_220 |
| Group 37 | 3 | 0.9999 | 0.9999 | MP_CBR91 | MP_CBR99 | MP_OV_2018_316 | MP_OV_2018_207, MP_OV_2018_316, MP_OV_2018_315 |
| Group 38 | 2 | 1 | 1 | #49 | *48 | MP_OV_2018_194 | MP_OV_2018_194, MP_OV_2018_287 |
| Group 39 | 2 | 1 | 0.9996 | #8 | *44 | MP_OV_2018_288 | MP_OV_2018_294, MP_OV_2018_288 |
| Group 40 | 2 | 1 | 0.9998 | #2 | *1 | MP_OV_2018_158 | MP_OV_2018_173, MP_OV_2018_158 |
