# Supplementary material for: Using multiple sources during reintroduction of a locally extinct population benefits survival and reproduction of an endangered freshwater fish
Source: Evol Appl. 2020 Dec 15;14(4):950–64. doi: 10.1111/eva.13173 (PMC8061264; doi:10.1111/eva.13173)
Supplement: Supplementary file 3 — Appendix S1 [file EVA-14-950-s003.zip › eva_13173_AppendixC_TR.docx]

**Appendix C. *Details of data and calculations for the offspring survival models***

Methods

To assess whether parental cross-type and genetic dissimilarity between broodstock parents can predict offspring survival in the Ovens River after stocking, we built models using genotypic data for 53 broodstock pairs bred in six consecutive breeding seasons from 2012/2013 to 2017/2018 and Ovens fish captured during monitoring and assigned as their offspring by parentage analyses. Only broodstock pairs with both parents genetically sampled were included in the survival model (Table A1); 2010/2011 breeding season was excluded, because it had only one broodstock pair genetically sampled. Broodstock pairs that produced larvae (and presumably contributed fingerlings to stocking) but had no offspring identified in Ovens were included in this analysis.

We first estimated the number of fingerlings produced by each pair that were stocked to the Ovens River (Table A1, tab **Fingerlings.csv** in Supporting Information 2) by dividing the number of larvae produced by that pair by the total number of larvae produced that year by all broodstock pairs (including those not genetically sampled), and multiplying it by the total number of fingerlings stocked that year into the Ovens (from Table A2).

For example, in 2013, a pair of broodstock fish from the Yarra River, a female MP_GBR62 and a male MP_GBR68 produced 12,000 larvae who grew into fingerlings. Given that a total of 134,035 larvae from 18 pairs bred in 2013 were produced (Table A1) and that the total number of fingerlings released into Ovens in 2014 (at age zero) was 40,500 (Table A2), this translates to the contribution of MP_GBR62/MP_GBR68 to the Ovens stocking being 3,626 fingerlings (Table A1).

For each pair, the minimum number of surviving offspring was calculated for each of eight age classes (from zero to 5 years old, 5 being the maximum age during 2018 monitoring season, for fish stocked in 2013; Table C1), based on the number of offspring assigned to each pair by parentage analyses for each year of sampling (from 2016 to 2018; Table B1). Offspring sampled in a particular age class were assumed to be present in all previous age classes.

For the example above for a broodstock pair MP_GBR62/MP_GBR68, among fish sampled in the Ovens in 2016, 2017 and 2018, six, three and one, respectively, were assigned to be their offspring (Table B1). Because only unique individuals were included, by the analogy with capture-recapture methods, the minimum number of surviving offspring for age classes 0, 1, 2, 3 and 4 were inferred to be 10, 10, 10, 4 and 1, respectively (Table C1).

Next, the relative probability of offspring survival for each age class was estimated as the minimum number of surviving offspring for each age class divided by the number of fingerlings of each pair that were stocked to the Ovens (Table A1).

We used the following R script to run Binomial survival models of stocked offspring. Input files are available in supporting Information 2).

# analysis of translocation outcomes for Macquarie perch (Macquaria australasica)

# as a function of genetic diversity and cross type

# load some packages

library(readxl)

library(lme4)

# load a helper function:

# assign broodstock pair to a cross type based on individual ID

determine_crosstype <- function(x) {

# GBR is Yarra, CBR is Dartmouth

is_dartmouth <- grepl("CBR", x)

is_yarra <- grepl("GBR", x)

# is it one or both?

cross_type <- ifelse(

is_dartmouth & is_yarra,

"DY",

ifelse(is_dartmouth, "DD", "YY")

)

# return

cross_type

}

# load the data (sheets in excel file)

survival_info <- read_xlsx("data/Supporting_Information_2_EVA_revised.xlsx",

sheet = "Survival_Model.csv")

num_fingerling <- read_xlsx("data/Supporting_Information_2_EVA_revised.xlsx",

sheet = "Fingerlings.csv")

survival_data <- read_xlsx("data/Supporting_Information_2_EVA_revised.xlsx",

sheet = "Number_alive.csv")

# remove ages 6 and 7 from the survival data

survival_data <- survival_data[, c("Broodstock_pair", "Age0", "Age1", "Age2", "Age3", "Age4", "Age5")]

# prepare survival data

# match number released with rows of num_alive

survival_data$Released <- num_fingerling$fingerlings[match(survival_data$Broodstock_pair, num_fingerling$Broodstock_pair)]

# calculate cohort ID and add to the full data set

cohort <- tapply(survival_info$Year_of_stocking, survival_info$Broodstock_pair, unique)

survival_data$Cohort <- cohort[match(survival_data$Broodstock_pair, names(cohort))]

# add genetic distance for each broodstock pair to full data set

gendist <- tapply(survival_info$Genetic_distance_percentage, survival_info$Broodstock_pair, unique)

survival_data$Genetic_distance <- gendist[match(survival_data$Broodstock_pair, names(gendist))]

# add cross type to num_alive data

survival_data$Crossing <- determine_crosstype(survival_data$Broodstock_pair)

# rescale variables for survival models

survival_data$Genetic_distance_std <- scale(survival_data$Genetic_distance)

# expand data out to a single column

count_tmp <- unlist(survival_data[, grep("Age", colnames(survival_data))])

age_tmp <- rep(0:5, each = nrow(survival_data))

survival_data <- do.call(

rbind,

lapply(1:6, function(x) survival_data)

)

survival_data$Survived <- as.numeric(count_tmp)

survival_data$Age <- age_tmp

# remove NA counts

survival_data <- survival_data[!is.na(survival_data$Survived), ]

# fit mixed model of growth residuals against a set of genetic and

# cross-type predictors

vars <- c("Crossing", "Genetic_distance_std", "(Genetic_distance_std + Crossing)")

vars <- c(vars, paste0("Age * ", vars))

vars <- paste0(vars, " + ")

vars <- c("", vars)

surv_form <- paste0("cbind(Survived, Released - Survived) ~ ", vars, "(1 | Cohort)")

surv_mod <- vector("list", length = length(surv_form))

for (i in seq_along(surv_form))

surv_mod[[i]] <- glmer(as.formula(surv_form[i]), data = survival_data, family = binomial)

# summarise the fitted models

surv_mod_summary <- lapply(surv_mod, summary)

# calculate r2 for each model

surv_mod_r2 <- lapply(surv_mod, MuMIn::r.squaredGLMM)

# AIC model comparison:

surv_mod_aic <- sapply(surv_mod, AIC)

Results

The survival model that included age class, genetic dissimilarity between members of a broodstock pair, and parental cross-type was the most parsimonious (lowest AIC) (Table 2 in the main manuscript and Table C2). Cross type and genetic dissimilarity were significantly associated with survival, with Yarra x Yarra broodstock pairs associated with the highest survival, followed by Dartmouth x Yarra pairs then Dartmouth x Dartmouth pairs (Table 2 in the main manuscript). Offspring from pairs with higher genetic dissimilarity had lower survival than those from less genetically dissimilar broodstock, conditional on the effects of cross type (i.e. more dissimilar broodstock produced worse-surviving offspring relative to other broodstock pairs of the same cross type; Table 2 in the main manuscript).

The negative within cross-type effect of parental dissimilarity was weak and inconsistent (Fig. 2d in the main manuscript). The following explains the meaning of the numerical coefficients (Table 2), based on the “linear predictor” (i.e. the fitted value on the logit scale). The mean estimates for each cross type are DxY = 1.62, YxY = 2.28 and DxD = 0 (model intercept). Based on the estimated effect of dissimilarity (-0.43), overriding the differences among cross types would require very large increases in genetic dissimilarity. For example, overcoming the 1.62 unit difference between a DxY pair and a DxD pair would require an increase in dissimilarity of 3.77 units (3.77 * -0.43 ~= -1.62), which is a change in dissimilarity of 3.77 standard deviations (SD of dissimilarity = 0.0091), or approximately 0.03. Overcoming the 0.66 unit difference between a YxY pair and a DxY pair (YxY – DxY = 2.28 – 1.62 = 0.66) would require an increase in dissimilarity of 1.53 units (1.53 * -0.43 ~= 0.66), which is a change in dissimilarity of 1.53 standard deviations, or approximately 0.014. Thus, the negative effect of dissimilarity is so small in magnitude that it would take an extreme change in dissimilarity to undo the effects of cross-type. Changes in dissimilarity of this magnitude would be similar to or greater than the differences among cross types. In other words, to outweigh the differences in survival among cross-types, dissimilarities would have to be extrapolated beyond the range of the observed values within each cross-type.

To ensure that significant relationships between cross-type, parental genetic dissimilarity and offspring survival is not driven by superior fit of the offspring of Yarra ancestry to the Ovens environments, we re-ran our analysis of survival after excluding Yarra x Yarra broodstock pairs. The same model structure was the most parsimonious, and included the same significant relationships between cross type, parental genetic dissimilarity and survival, despite lower sample sizes (Table C3).

**Table C1** Per broodstock pair calculations of minimum number of stocked offspring surviving to different ages for 53 pairs of broodstock fish that yielded fingerlings stocked to the Ovens River and had genetic samples analysed for both members of a pair.

| Broodstock pair | Age0 | Age1 | Age2 | Age3 | Age4 | Age5 |
| --- | --- | --- | --- | --- | --- | --- |
| MP_CBR03/MP_CBR10 | 0 | 0 | 0 | 0 | 0 | 0 |
| MP_GBR01/MP_GBR07 | 1 | 1 | 1 | 1 | 1 | 1 |
| MP_GBR02/MP_GBR11 | 0 | 0 | 0 | 0 | 0 | 0 |
| MP_GBR03/MP_GBR08 | 2 | 2 | 2 | 2 | 2 | 2 |
| MP_GBR04/MP_GBR09 | 1 | 1 | 1 | 1 | 1 | 1 |
| MP_GBR05/MP_GBR10 | 1 | 1 | 1 | 1 | 1 | 0 |
| MP_CBR54/MP_CBR69 | 0 | 0 | 0 | 0 | 0 | NA |
| MP_CBR55/MP_CBR78 | 0 | 0 | 0 | 0 | 0 | NA |
| MP_CBR56/MP_CBR70 | 0 | 0 | 0 | 0 | 0 | NA |
| MP_CBR59/MP_CBR71 | 0 | 0 | 0 | 0 | 0 | NA |
| MP_CBR60/MP_CBR73 | 1 | 1 | 1 | 0 | 0 | NA |
| MP_CBR61/MP_CBR72 | 0 | 0 | 0 | 0 | 0 | NA |
| MP_CBR62/MP_CBR74 | 0 | 0 | 0 | 0 | 0 | NA |
| MP_CBR63/MP_CBR75 | 0 | 0 | 0 | 0 | 0 | NA |
| MP_CBR64/MP_CBR77 | 0 | 0 | 0 | 0 | 0 | NA |
| MP_CBR67/MP_CBR76 | 3 | 3 | 3 | 1 | 0 | NA |
| MP_GBR05/MP_GBR08 | 0 | 0 | 0 | 0 | 0 | NA |
| MP_GBR30/MP_GBR51 | 0 | 0 | 0 | 0 | 0 | NA |
| MP_GBR62/MP_GBR68 | 10 | 10 | 10 | 4 | 1 | NA |
| MP_GBR64/MP_GBR70 | 3 | 3 | 3 | 3 | 2 | NA |
| MP_GBR65/MP_GBR69 | 0 | 0 | 0 | 0 | 0 | NA |
| MP_GBR80/MP_GBR79 | 0 | 0 | 0 | 0 | 0 | NA |
| MP_CBR91/MP_CBR99 | 3 | 3 | 3 | 3 | NA | NA |
| MP_CBR91/MP_GBR14 | 13 | 13 | 8 | 7 | NA | NA |
| MP_CBR92/MP_CBR100 | 5 | 5 | 4 | 3 | NA | NA |
| MP_CBR92/MP_GBR09 | 16 | 16 | 11 | 3 | NA | NA |
| MP_CBR93/MP_CBR101 | 7 | 7 | 4 | 3 | NA | NA |
| MP_CBR93/MP_GBR67 | 2 | 2 | 2 | 2 | NA | NA |
| MP_CBR94/MP_CBR102 | 0 | 0 | 0 | 0 | NA | NA |
| MP_CBR94/MP_GBR60 | 4 | 4 | 2 | 1 | NA | NA |
| MP_CBR95/MP_CBR103 | 1 | 1 | 1 | 1 | NA | NA |
| MP_CBR95/MP_GBR53 | 2 | 2 | 1 | 0 | NA | NA |
| MP_CBR96/MP_GBR79 | 4 | 2 | 1 | NA | NA | NA |
| MP_CBR97/MP_GBR08 | 4 | 3 | 2 | NA | NA | NA |
| MP_CBR98/MP_CBR77 | 5 | 2 | 2 | NA | NA | NA |
| MP_CBR139/MP_CBR153 | 1 | 1 | NA | NA | NA | NA |
| MP_CBR140/MP_CBR155 | 0 | 0 | NA | NA | NA | NA |
| MP_CBR141/MP_CBR158 | 1 | 1 | NA | NA | NA | NA |
| MP_CBR142/MP_CBR154 | 0 | 0 | NA | NA | NA | NA |
| MP_GBR30/MP_CBR156 | 0 | 0 | NA | NA | NA | NA |
| MP_CBR1390/MP_CBR1402 | 0 | NA | NA | NA | NA | NA |
| MP_CBR1391/MP_CBR1403 | 0 | NA | NA | NA | NA | NA |
| MP_CBR1392/MP_CBR1404 | 0 | NA | NA | NA | NA | NA |
| MP_CBR1393/MP_CBR1405 | 1 | NA | NA | NA | NA | NA |
| MP_CBR1394/MP_CBR1406 | 0 | NA | NA | NA | NA | NA |
| MP_CBR1395/MP_CBR1407 | 0 | NA | NA | NA | NA | NA |
| MP_CBR1396/MP_CBR1408 | 0 | NA | NA | NA | NA | NA |
| MP_CBR1397/MP_CBR1407 | 0 | NA | NA | NA | NA | NA |
| MP_CBR1398/MP_CBR1409 | 0 | NA | NA | NA | NA | NA |
| MP_CBR1399/MP_CBR1410 | 0 | NA | NA | NA | NA | NA |
| MP_CBR1400/MP_CBR1411 | 1 | NA | NA | NA | NA | NA |
| MP_CBR1401/MP_CBR103 | 0 | NA | NA | NA | NA | NA |
| MP_GBR30/MP_CBR1402 | 0 | NA | NA | NA | NA | NA |
| **Number of pairs per age class** | 53 | 40 | 35 | 32 | 22 | 6 |
| **Minimum number of offspring observed per age class** | 92 | 84 | 63 | 36 | 8 | 4 |

**Table C2.** Results of model comparison of survival model with genetic dissimilarity and cross-type. Year of stocking was included in all models as a random factor. Models were fitted with all data and with a reduced data set that excluded Yarra x Yarra (YxY) pairs. Model comparison was based on Akaike’s Information Criterion (AIC). Models are arranged in the order of descending AIC, the lowest AIC value (most parsimonious model) is in bold.

| **Included variables** | **AIC** | **AIC without YxY pairs** |
| --- | --- | --- |
| Intercept-only (null) model | 623.4 | 381.34 |
| Genetic dissimilarity | 592.95 | 371.06 |
| Age, genetic dissimilarity | 572.89 | 353.6 |
| Cross type | 551.98 | 351.89 |
| Genetic dissimilarity, cross type | 546.19 | 346.98 |
| Age, cross type | 531.93 | 334.25 |
| Age, genetic dissimilarity, cross type | **528.17** | **331.48** |

**Table C3.** Results of the most parsimonious survival model fitted to a reduced data set that excluded Yarra x Yarra (YxY) pairs: probability of offspring survival after stocking for pairs of broodstock fish as a function of age class, cross type, and genetic dissimilarity between members of a parent pair (GD). Year of stocking was fitted in all models as a random factor. R2m is a likelihood ratio-based pseudo R^2^ measure calculated with the R package MuMIn, using theoretically derived binomial variances. Significant P-values are in bold. Results of the full model are presented as Table 2 of the main manuscript.

|  |  | **Model without** YxY **pairs** | | | |  |
| --- | --- | --- | --- | --- | --- | --- |
| Variables | Cross type | Coefficient | Standard error | Pr(>\|z\|) | R2m  (theoretical) | |
| Intercept |  | –8.15 | 0.56 | **<0.001** | 0.22 | |
| Age class |  | –0.22 | 0.13 | 0.084 |  | |
| Genetic dissimilarity |  | –0.45 | 0.21 | **0.033** |  | |
| Cross type | DY | 1.64 | 0.38 | **<0.001** |  | |
|  | YY | NA | NA | NA |  | |
| Age class x genetic dissimilarity |  | 0.04 | 0.13 | 0.767 |  | |
| Age class x cross-type | DY | –0.14 | 0.23 | 0.546 |  | |
|  | YY | NA | NA | NA |  | |
